# Perforin-2 is a pore-forming effector of endocytic escape in cross-presenting dendritic cells

**DOI:** 10.1126/science.adg8802

**Published:** 2023-06-22

**Authors:** Pablo Rodríguez-Silvestre, Marco Laub, Patrycja A. Krawczyk, Alexandra K. Davies, Julia P. Schessner, Reejuana Parveen, Benjamin J. Tuck, William A. McEwan, Georg H.H. Borner, Patrycja Kozik

**Affiliations:** 1 MRC Laboratory of Molecular Biology; Cambridge, UK; 2 Department of Proteomics and Signal Transduction, Max Planck Institute of Biochemistry; Martinsried, Germany; 3 Current: School of Biological Sciences, Faculty of Biology, Medicine and Health, Manchester Academic Health Science Centre, University of Manchester, Manchester, UK; 4 UK Dementia Research Institute at the University of Cambridge, Department of Clinical Neurosciences; Cambridge, UK

## Abstract

During initiation of antiviral and antitumour T cell-mediated immune responses, dendritic cells (DCs) cross-present exogenous antigens on MHC class I. Cross-presentation relies on the unique “leakiness” of endocytic compartments in DCs, whereby internalised proteins escape into the cytosol for proteasome-mediated generation of MHC-I-binding peptides. Given that type 1 conventional DCs excel at cross-presentation, we searched for cell-type specific effectors of endocytic escape. We devised an assay suitable for genetic screening and identified a pore-forming protein, perforin-2 (*Mpeg1*), as a dedicated effector exclusive to cross-presenting cells. Perforin-2 was recruited to antigen-containing compartments, where it underwent maturation, releasing its pore-forming domain. *Mpeg1^-/-^* mice failed to efficiently prime CD8+ T cells to cell-associated antigens, revealing an important role for perforin-2 in cytosolic entry of antigens during cross-presentation.

The integrity of endosomal and lysosomal membranes is critical to protect the cell against extracellular pathogens, toxins, and from the activity of lysosomal hydrolases. In dendritic cells (DCs), however, internalised proteins are delivered from endocytic organelles into the cytosol where they can be proteolytically processed for presentation on MHC class I molecules (1). The ability of DCs to present exogenous peptides on endogenous MHC-I is termed cross-presentation. Cross-presentation is critical for initiation of cytotoxic T cell (CTL) responses to antigens not expressed in DCs such as neoantigens or antigens from virally infected cells (2–4).

Various mechanisms have been suggested to facilitate endocytic escape and promote cross-presentation (5–10). Early studies proposed that escape is mediated by protein channels (such as Sec61) recruited from the endoplasmic reticulum to endosomes (7, 11). More recent data suggest that escape occurs through unrepaired damage to membranes (e.g., due to ROS-driven lipid peroxidation) (6, 9, 10). Both models imply that the unusual “leakiness” of endocytic compartments in DCs might not rely on any cell type-specific effectors, but on the regulation of ubiquitously expressed proteins through signalling (12, 13) and trafficking (14) events unique to cross-presenting cells.

Here, to identify DC-specific regulators of endocytic escape, we developed a flow cytometry-based assay to monitor escape in individual cells, and applied it in a CRISPR/Cas9-based screen using cells specialised in cross-presentation, conventional DC1s (cDC1).

## A CRISPR/Cas9 screen identifies *Mpeg1* (perforin-2) as a regulator of endocytic escape

**Fig. 1:**
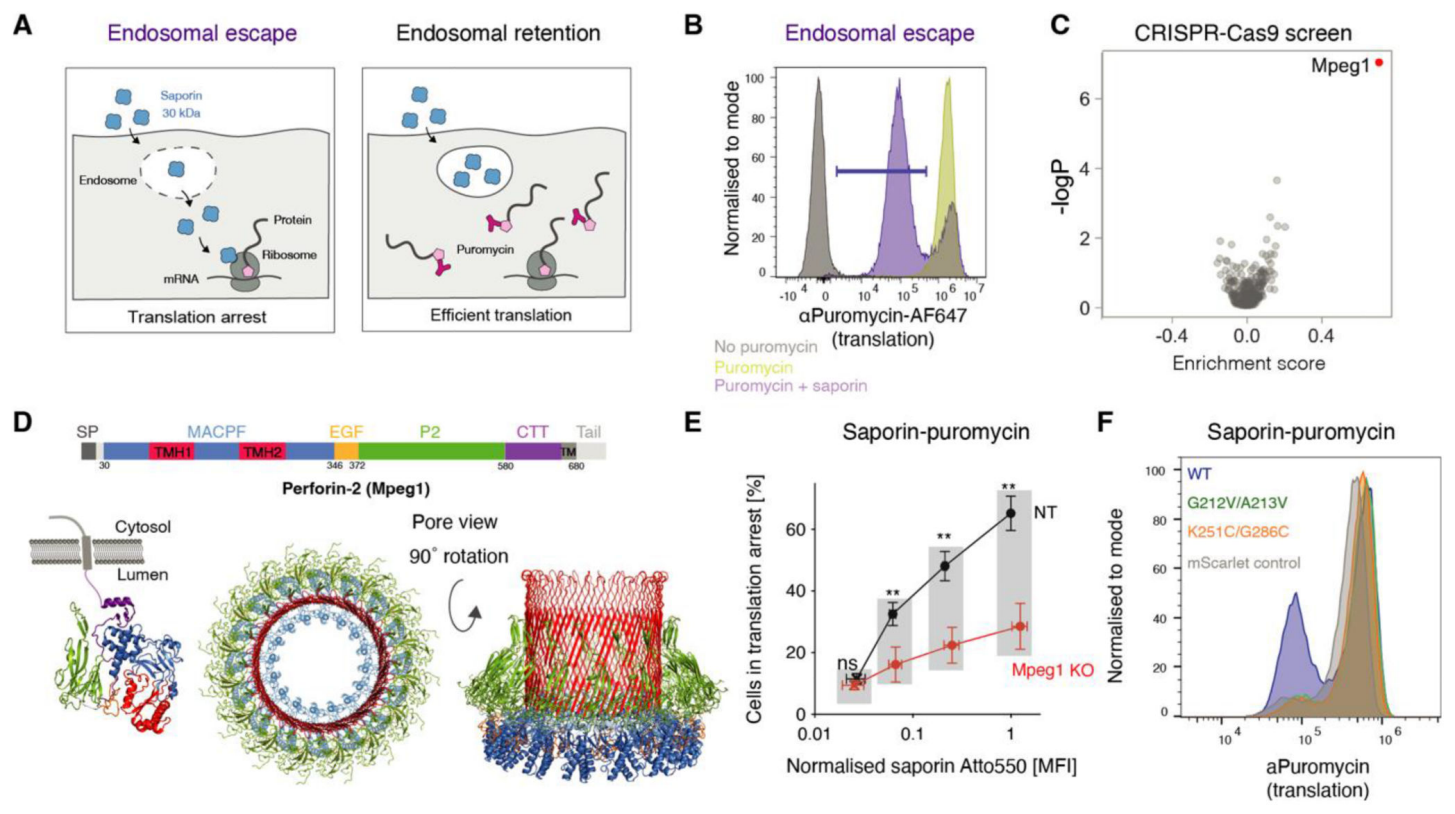
Saporin-puromycin assay to monitor endocytic escape in DCs. (A, B) Saporin-puromycin assay. (A) Schematic representation. Saporin-pulsed cells are labelled nascent puromycin to monitor translation rate. Puromycin incorporated into nascent peptides is detected with an **α**Puromycin Ab and flow cytometry. If saporin is retained within the endosomes, translation remains high. When saporin escapes into the cytosol it depurinates ribosomes inducing translation arrest. (B) Representative flow cytometry plot MutuDCs were incubated with 0.5 mg/ml of saporin followed by 0.01 mg/mL puromycin (purple histogram), with puromycin alone (yellow) or in media only (grey). Cells in translation arrest are denoted by the purple gate. See also fig. S1B. (C) Volcano plots showing the sgRNAs enrichment analysis for the saporin-puromycin endocytic escape screen. Each of the dots represents one targeted gene. Data represent the combined mean enrichment scores and the non-adjusted p values from three independent experiments (Fisher’s method). See also fig. S3. (D) Schematic representation of the different perforin-2 domains alongside structures of single subunit and hexadecameric perforin-2 in pre-pore (PDB ID: 6U2K and PDB ID: 6SB3) and pore-forming conformations (PDB ID: 6SB5). (E) Quantification of translation arrest in *Mpeg1^KO^* and NT MutuDCs. Cells were pulsed with saporin (11:1 unlabelled:Atto550-labelled saporin) for 2 h, and translation was monitored by a 30 min puromycin chase. The X axis represents Atto550 MFI normalised to the NT MutuDC Atto550 MFI at the highest saporin concentration. Data represent mean and SEM of three independent experiments, ns, not significant; *P<0.5; ** *P*<0.01; *** *P*<0.001; ****P<0.0001 using a multiple unpaired t-test (two-stage step-up, Benjamini, Krieger and Yekutieli). Significance symbols in the plot refer to the differences in proportion of cells in translation arrest. Differences in saporin Atto550 MFI were not significant. See also fig. S4A. (F) *Mpeg1*^KO^ MutuDCs were reconstituted with the indicated *Mpeg1* mutants or with mScarlet only and used in the saporin-puromycin assay with a 2 h pulse at 0.1 mg/ml saporin. Data are representative of three independent experiments. See also fig. S4C.

To monitor endocytic escape, we used the 28 kDa type I ribosome inactivating protein (RIP) saporin (5). Once in the cytosol, RIPs arrest translation through depurination of the sarcin-ricin loop in the 28S subunit of the ribosome (15). While type II RIPs, such as ricin, comprise a domain that facilities entry into the cytosol, cytosolic delivery of type I RIPs is dependent on the cell-intrinsic efficiency of endosome-to-cytosol transport (5). To detect saporin-induced translation inhibition, we monitored incorporation of puromycin, a structural analogue of aminoacyl tRNAs, into nascent polypeptides (16). We labelled intracellular puromycylated polypeptides with a fluorescent antibody 12D10 allowing for flow cytometry-based readout of translation efficiency and thereby of endocytic escape (Fig. 1A).

cDC1s, a subset of DCs that excels at cross-presentation in vivo (17), have been reported to have the most efficient endocytic escape pathway (17, 18). We thus developed the saporin-puromycin assay using a murine cDC1-like cell line, MutuDCs (13, 19, 20) (Fig. 1B and fig. S1, A and B). We confirmed that saporin escape is the rate-limiting step in the assay (fig. S1, C and D) and that the assay recapitulates previously reported differences in escape efficiency, namely more efficient escape in cDC1s compared to cDC2s (fig. S2, A and C) (18, 21) and enhanced “leakiness” of endocytic compartments in cells from lupus-prone MRL/MpJ-Fas^lpr^/J mice (fig. S2, B and D) (22).

We then employed the saporin-puromycin assay in a CRISPR/Cas9-based screen of 281 genes highly expressed in cDC1s compared to cDC2s (fig. S3). We used a mix of Atto550-labelled and unlabelled saporin, gated on cells with similar uptake efficiency, and sorted them into two bins: puro^low^ (saporin escape, translation arrest) and puro^high^ (saporin retention, efficient translation) (fig. S3C). In the absence of saporin, none of the sgRNAs affected the translation rate (fig. S3E, table S1). The strongest hit in saporin-pulsed cells was *Mpeg1* (Fig. 1C), with the four sgRNAs enriched in puro^low^ vs puro^high^ populations (fig. S3F).

## Perforin-2 is necessary for endocytic escape in DCs

*Mpeg1* encodes perforin-2, a member of the membrane attack complex (MAC) and perforin superfamily (MACPF) of pore-forming proteins (23). Perforin-2 can form oligomeric pores on liposomes with an opening of at least 75 Å (24–26) (Fig. 1D). It was initially proposed that perforin-2 pores facilitate killing of intravacuolar bacteria (27), but these results were not replicated in a recent study (28). Here, we explored whether perforin-2 can function as an effector of endocytic escape.

We generated *Mpeg1*^KO^ MutuDCs and confirmed protein depletion in sorted, sgRNA-expressing BFP^+^ cells (fig. S4A). To account for the effect of passage numbers on MutuDC behaviour (20), we cultured control cells expressing non-targeting (NT) sgRNA in parallel with the KO line. We first demonstrated that disruption of *Mpeg1* protected the cells from the cytotoxic effects of saporin and from death induced by a different RIP, gelonin (fig. S5A). We also tested the sensitivity of *Mpeg1^KO^* DCs to a glycopeptide chemotherapeutic, bleomycin A2, which induces DNA damage, but due to its hydrophilicity does not enter the cells efficiently (29). We used automated imaging to monitor MutuDC growth rate and found that loss of perforin-2 rendered the cells more resistant to bleomycin-mediated cytotoxicity (fig. S5A). Importantly, the *Mpeg1^KO^* cells were not protected against the effects of poly(I:C), which induces cell death via endosomal Toll-like receptor 3 (TLR3) (30), or against membrane-permeable cycloheximide (fig. S5A).

We next verified that the sensitivity of perforin-2-expressing DCs to cytosolic toxins is due to efficient escape rather than due to inefficient uptake. In the saporin-puromycin assay with Atto550-labelled saporin, endocytic escape was impaired in the *Mpeg1*^KO^ DCs, even though they internalised similar amounts of saporin to NT cells (Fig. 1E). The differences in escape were also not due to changes in DC activation, because neither NT nor *Mpeg1*^KO^ MutuDCs were activated by saporin (fig. S5B). Finally, we rescued saporin import without affecting uptake by transducing *Mpeg1*^KO^ cells with full length sgRNA-resistant *Mpeg1* (fig. S4C and fig. S5C).

Next, we addressed whether the pore-forming ability of perforin-2 is required for endocytic escape. Perforin-2 forms pores by oligomerisation and unwinding of two helices in the MACPF domain into β sheets (in red in Fig. 1D) (24, 25). To test whether this conformational change is required for endocytic escape, we generated two mutants: *Mpeg1*^G212V/A213V^ with mutations in the conserved MACPF motif (31) and *Mpeg1*^K251C/G286C^, with a disulphide bond known to constrain one of the pore-forming helices preventing pore formation in vitro (24). Both, *Mpeg1*^G212V/A213V^ and *Mpeg1*^K251C/G286C^, were expressed at similar levels to Mpeg1^*WT*^, but neither rescued saporin escape in *Mpeg1*^KO^ MutuDCs (Fig. 1F and fig. S4C). Thus, perforin-2 pores mediate endocytic escape in cross-presenting DCs.

## Perforin-2 is sufficient for endocytic escape in non-immune cells

Because perforin-2 expression is restricted to antigen presenting cells (32, 33), we asked whether it is sufficient for endocytic escape in non-immune cells. We generated HEK293T and HeLa cells co-expressing murine *Mpeg1* with *mScarlet* or *BFP*, or expressing the fluorescent protein alone, and used them in a range of escape assays.

**Fig. 2:**
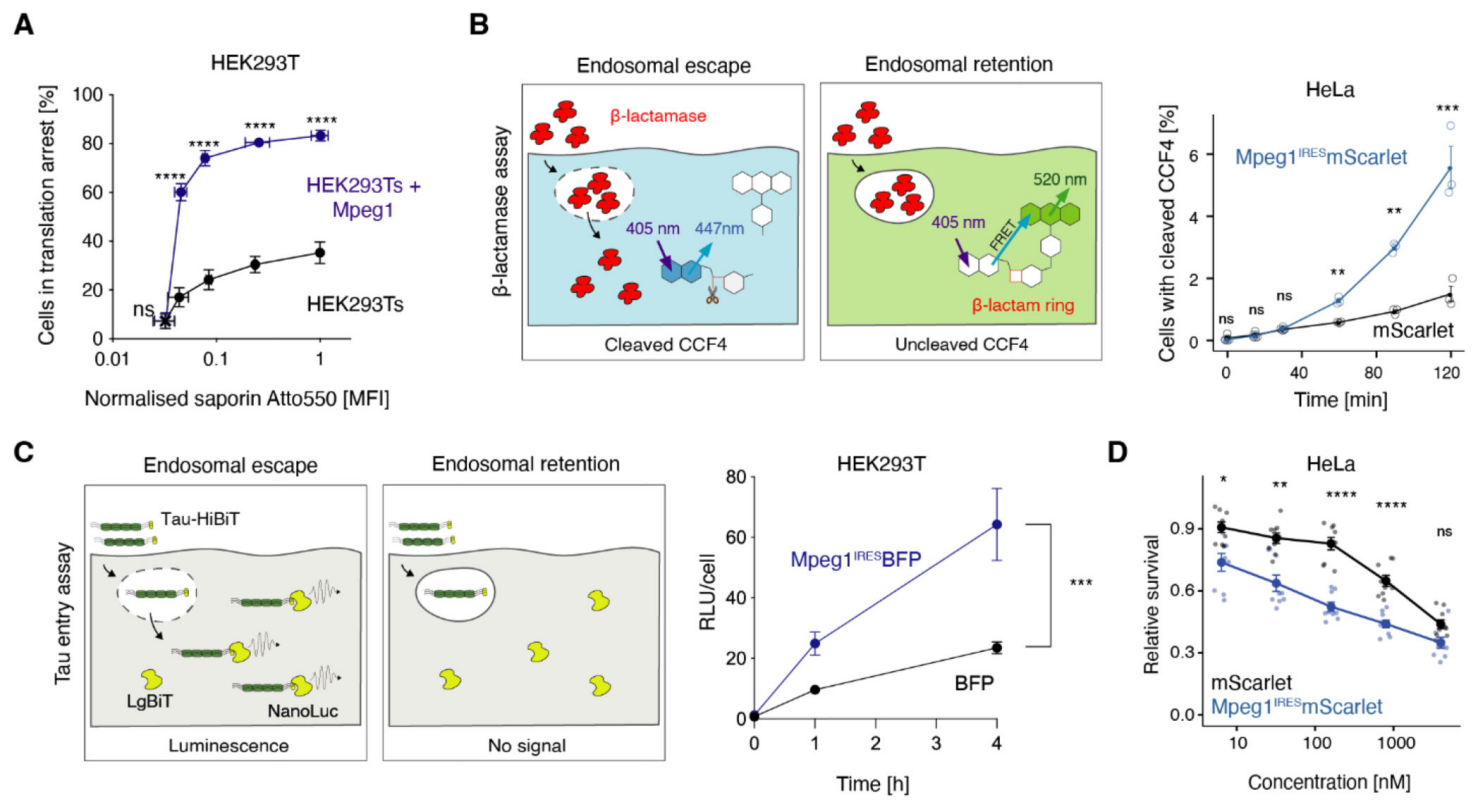
Perforin-2 is sufficient for endocytic escape of cargo in non-immune cells. (A) HEK293Ts and Mpeg1-complemented HEK293Ts were pulsed with saporin (11:1 unlabelled:Atto550-labelled saporin) for 2 h, and translation was monitored by a 30 min puromycin chase. The x-axis represents Atto550 MFI normalised to the WT cells pulsed with 0.5 mg/ml saporin. Data represent mean and SEM of three independent experiments, ns, not significant; * P<0.5; **P<0.01; ***P<0.001’ ****P<0.0001 using a multiple t-test (Bonferroni-Dunn). Significance symbols on the plot refer to the differences in cells in translation arrest. Differences in saporin Atto550 MFI were not significant. (B) **Cytosolic escape of β**-lactamase in cells loaded with the CCF4 results in CCF4 cleavage, loss of FRET and shift in emission fluorescence. HeLas expressing either *Mpeg1*^IRES^-mScar let or mScarlet only were pulsed with β-lactamase for the indicated time. β-lactamase escape was monitored by measuring the shift in fluorescence emission by flow cytometry. Data represent mean and SEM of three independent experiments, ns, not significant, *P<0.5; **P<0.01; ***P<0.001; ****P<0.0001 using a multiple t-test (two-stage step-up, Benjamini, Krieger and Yekutieli) For gating strategy see fig. S6. (C) To monitor the escape of Tau oligomers, cells expressing NLS-eGFP-LargeBiT were pulsed with Tau-HiBit oligomers. The escape of Tau-HiBiT into the cytoplasm allows binding to the 18 kDA luciferase subunit, LgBiT. This results in reconstitution of catalytic activity and generation of luminescence. NLS-eGFP-LargeBiT HEK293Ts expressing either *Mpeg1*^IRES^-BFP or BFP-only were pulsed with tau-HiBiT for the indicated time. Following substrate addition, luminesce and cell viability were assessed. Relative luminescent units (RLUs) were then normalised to viability per well. Data represent mean and SEM of three independent experiments each with six technical replicates, ***P<0.001 using a paired t-test. (D) HEK293Ts were plated in the presence or absence of bleomycin and cultured in an Incucyte ® for 48 h to monitor the growth rate. Data represent mean and SEM of three independent experiments each with four wells per condition, ns, not significant; *P<0.5; **P<0.01; ***P<0.001; ****P<0.0001 using a multiple t-test (Bonferroni-Dunn).

Ectopic expression of perforin-2 was sufficient to promote saporin escape in HEK_293_Ts (Fig. 2A). We also monitored escape of 34 kDa β-lactamase using a cytosolic dye CCF_4_ (34, 35). CCF_4_ consists of fluorescein and 7-hydroxycoumarin linked via a β-lactam ring, which is cleaved when β-lactamase escapes into the cytosol, resulting in a shift in fluorescence emission (Fig 2B). Expression of perforin-2 in HeLa cells increased the frequency of cells with cleaved CCF4 and thus the efficiency of β-lactamase escape (Fig. 2B and fig. S6). We then adopted a split luciferase-based assay to monitor endocytic escape of microtubule-associated protein, tau (36). We expressed the large 18 kDa NanoLuc subunit (LgBiT) in the cytosol of HEK293Ts and pulsed the cells with oligomers of 42 kDa tau fused to the short HiBiT peptide. Upon entry into the cytosol, LgBiT binds HiBiT resulting in catalytically active luciferase and luminescence in the presence of the Nano-Glo^(R)^ substrate. Again, in perforin-2-expressing HEK293Ts, tau-HiBit escaped more efficiently compared to the control line (Fig. 2C). Finally, we demonstrated that expression of perforin-2 renders HeLa cells more sensitive to bleomycin-mediated toxicity (Fig. 2D). Thus, ectopic expression of perforin-2 is sufficient to drive endocytic escape in non-immune cells.

## Perforin-2 is proteolytically processed in lysosomes

Given the cytotoxic potential of pore-forming proteins, we asked how cDC1s regulate pore-formation and restrict it to antigen containing compartments (19, 36). Perforin-2 is the only known mammalian pore-forming protein with an additional transmembrane domain (TMD) not involved in pore formation (see Fig. 1D). The TMD has been proposed to act as an anchor preventing damage to endogenous membranes and orientating the pore towards intravacuolar bacteria (24, 27). We hypothesised that the ectodomain would have to be proteolytically released to facilitate endocytic escape.

**Fig. 3:**
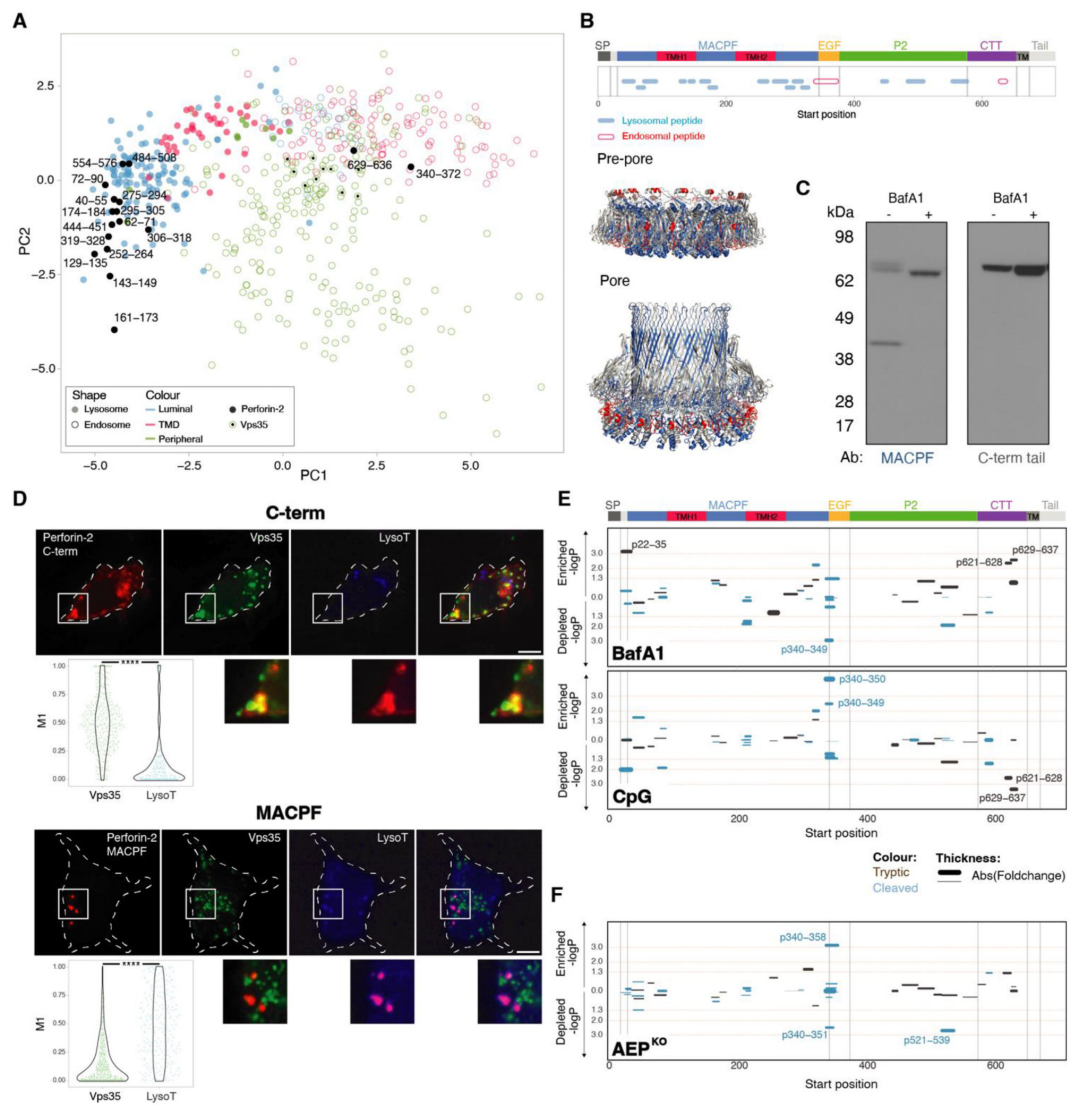
Perforin-2 undergoes proteolytic cleavage releasing its pore forming domain into the organellar lumen. (A, B) (A) Principal component analysis of mass spectrometry-based organellar mapping of MutuDCs (19) (see also fig. S7A). The maps were prepared from control MutuDCs and cells treated with drugs that promote lysosomal leakiness, prazosin and tamoxifen. Peptides derived from lysosomal and endosomal proteins are represented by filled and empty circles, respectively. The different colours indicate localisation of the protein within the corresponding organelle. Perforin-2 peptides are displayed as filled black circles regardless of their localisation. (B) Mapping of the different lysosomal and endosomal perforin-2 peptides detected by organellar mass spectrometry onto the different perforin-2 domains and structure. (C) Perforin-2 levels in NT MutuDCs treated with 0.5 **μ**M BafA1 for 3 h were assessed by immunoblot under reducing conditions using the **α**MACPF and **α**C-terminal tail antibodies. (D) Confocal microscopy images of MutuDCs stained for perforin-2 with either **α**C-terminal tail or **α**MACPF antibodies (red), Vps35 (green) and lysotracker (blue). Data represent two independent experiments each with at least 80 cells, ***P<0.0001 using a Kolmogorov-Smirnov test. (E) BafA1 and CpG induced changes in the abundance of tryptic and semi-tryptic (cleaved) perforin-2 peptides. Control and treated cells (1 μM BafA1 or 1 **μ**M CpG) were analysed by mass spectrometry, and peptide intensities were normalised to corresponding protein intensities. Statistical analysis was performed with a two-sided student’s t-test. *P*-values (y-axis) and fold change in abundance (line thickness) in treated vs control cells are shown. The amino acid position indicates the location of the peptides along the different perforin-2 domains. (F) Differences in the abundance of tryptic and semi-tryptic (cleaved) perforin-2 peptides between control and *AEP*^KO^ MutuDCs (fig. S9B) by full proteome mass spectrometry. The analysis was performed as in (E).

To investigate the proteolytic processing of perforin-2, we analysed a SILAC-based organellar mapping dataset generated previously (19). The maps were prepared by mass spectrometry-based analysis of the fractionated post-nuclear supernatants from MutuDCs (fig. S7A) (37). In the original maps, perforin-2 had a profile similar to lysosomal proteins. Here, instead of analysing protein profiles, we analysed each tryptic peptide individually, only including endo- or lysosomal proteins (Fig. 3, A and B). Peptides derived from endosome- and lysosome-resident proteins formed separate clusters, as did peptides derived from transmembrane and luminal proteins in the lysosome cluster. 15 out of 17 perforin-2-derived peptides clustered with soluble rather than transmembrane lysosomal proteins consistent with the hypothesis that the perforin-2 ectodomain is released from the TMD anchor. The remaining perforin-2 peptides, p340-372 (within the EGF domain) and p629-635 (within the TMD-proximal region), co-clustered with endosomal proteins (such as Vps35, Fig. 3A) suggesting that they are present in the full-length protein transiting through endosomes but are absent (cleaved) once perforin-2 reaches lysosomes.

We confirmed that perforin-2 was proteolytically processed using antibodies against the MACPF, P2 and the C-terminal tail (C-term) (fig. S7, B and C). All three detected full-length perforin-2 at 70 kDa. We also identified a 40 kDa MACPF and a 30 kDa P2 fragment, consistent with the cleavage in the EGF domain. The αC-term antibody detected only the full-length protein indicating that the tail (and most likely the TMD) are rapidly degraded following release of the ectodomain. Bafilomycin A1, a V-ATPase inhibitor that interferes with lysosomal acidification, accumulated full-length perforin-2, consistent with the hypothesis that proteolytic processing occurs in lysosomes (Fig. 3C). Finally, confocal microscopy confirmed that the αC-term antibody, predicted to recognise the immature (full-length) perforin-2, co-localised with Vps35 (Fig. 3D), and the αMACPF antibody colocalised with lysotracker, but not with Vps35. This together with the organellar mapping data suggests that the majority of the protein is in lysosomes at steady-state. Thus, full-length perforin-2 resides in (or transits through) endosomes, and upon reaching low-pH compartments, undergoes maturation involving at least two cleavage events.

## Perforin-2 maturation is controlled by Asparagine endopeptidase (AEP)

We then asked whether perforin-2 maturation might be regulated by antigen-associated signals. From a panel of TLR agonists, CpG (TLR9-agonist) and *Toxoplasma* profilin (TLR11-agonist) were most efficient in promoting perforin-2 proteolytic processing (fig. S8). To further characterise the putative cleavage sites, we performed comparative proteomics analysis of tryptic and semi-tryptic peptides from CpG-, BafA1- and mock-treated cells (Fig. 3E). Semi-tryptic peptides are considered to be derived from proteins that were cleaved in the cell, prior to sample processing. The semi-tryptic peptide p340-349 (within the EGF domain) was significantly enriched in CpG-treated cells and depleted in BafA1-treated cells, while tryptic peptides near the TMD, p629-628 and p629-637, were depleted in CpG-treated cells and enriched in BafA1-treated cells.

Several of the non-tryptic peptides in the EGF region terminated on asparagine (fig. S9A) suggesting that perforin-2 processing might be mediated by AEP (38). Indeed, the cleavage pattern in the EGF domain was different in *AEP*^KO^ MutuDCs (fig. S9B) compared to control cells (see peptides p340-358 and p340-351, Fig. 3F), suggesting that AEP mediates perforin-2 maturation, but its activity can be replaced by other enzymes (most likely cathepsins (39)). Immunoblot analysis confirmed that the EGF cleavage is less efficient (albeit not completely abolished) in the absence of AEP, resulting in accumulation of the 60 kDa ectodomain (fig. S9C). Finally, we were also able to reconstitute this cleavage in vitro using recombinant perforin-2 and AEP (fig. S9D).

In summary, perforin-2 maturation is controlled by at least two cleavage events, both occurring at steady-state but stimulated by TLR signalling. The cleavage in the TMD-proximal CTT domain releases the ectodomain into the lysosomal lumen to orient the pore-forming domain towards the endogenous membranes. The AEP-mediated cleavage in the EGF region, which is not required for pore formation in vitro (24, 40), may provide additional flexibility to complete pore insertion in vivo or may serve to inactivate the pores.

## Perforin-2 undergoes maturation in antigen-containing compartments

To test whether perforin-2 undergoes maturation upon recruitment to antigen-containing compartments or whether antigen-containing compartments acquire mature perforin-2, we analysed perforin-2 processing in phagosomes.

**Fig. 4:**
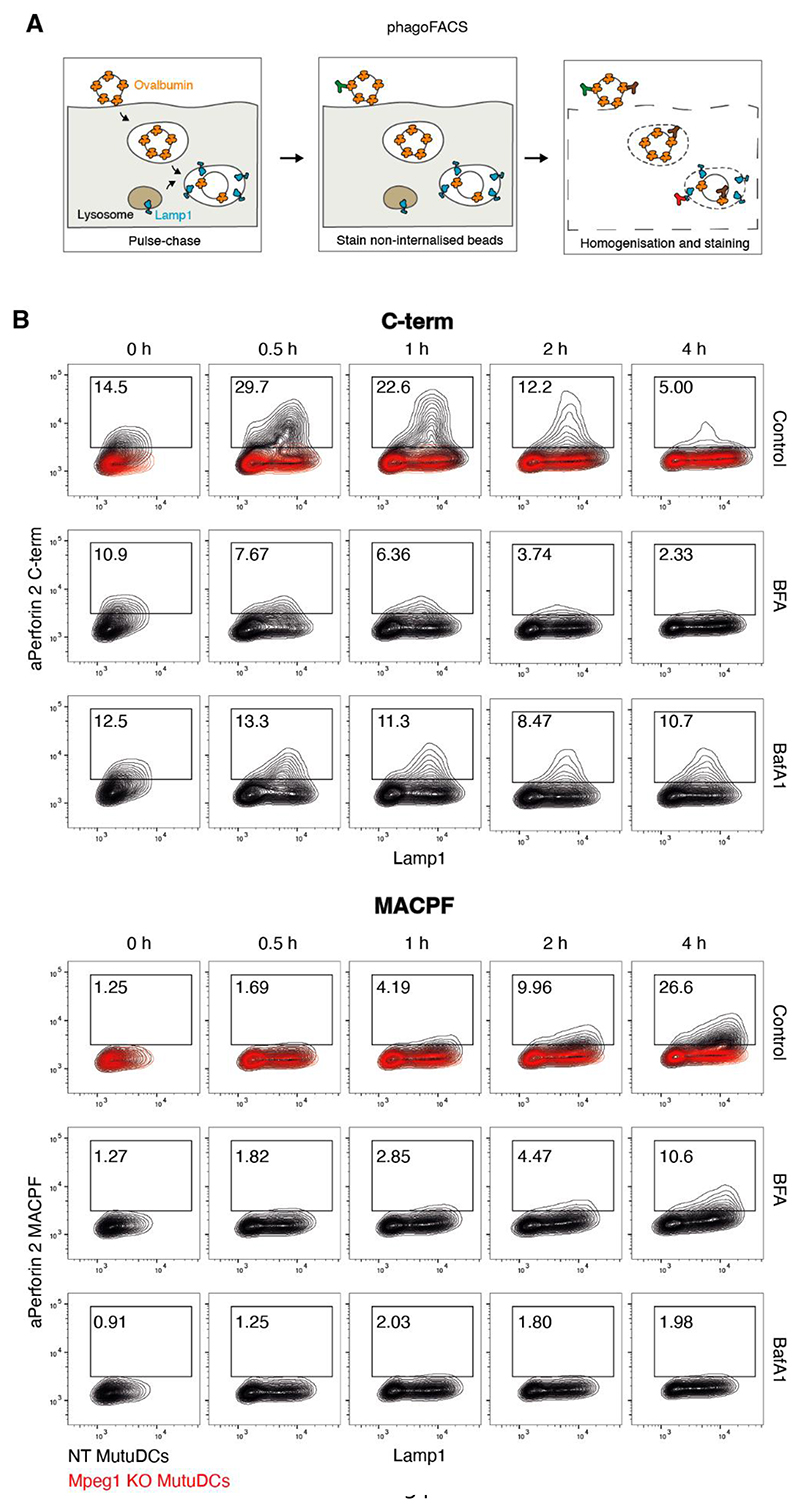
Perforin-2 undergoes pH-dependent maturation in antigen-containing phagosomes. (A) Schematic representation of the phagoFACS assay. Cells are pulsed with OVA-beads and allowed to internalise them. After an indicated chase period, non-internalised beads are marked with an α Ovalbumin antibody. Following cell homogenisation, phagosomes are stained with antibodies against ovalbumin coupled to an alternative fluorophore and phagosomal markers. (B) *Mpeg1^KO^* and NT MutuDCs were pulsed with OVA-beads and chased for the indicate time in the presence of either BFA or BafA1. Isolated phagosomes were stained with antibodies against Lamp1 and either the perforin-2 C-terminal tail (top panel) or MACPF domain (bottom panel). Data are representative of three independent experiments. See also fig. S11 for gating strategy, quantification, and additional plots.

We first confirmed that perforin-2 mediates the escape of bead-conjugated saporin from phagosomes into the cytosol (fig. S10). We then followed perforin-2 maturation in individual phagosomes by phagoFACS, a technique in which DCs are pulsed with ovalbumin-coated beads and the resulting phagosomes are analysed by flow cytometry (Fig. 4A). Using the αC-term antibody, we demonstrated that perforin-2 was rapidly recruited to phagosomes reaching its highest levels within 30 min (Fig. 4B and fig. S11, A and B). This rapid acquisition suggests that perforin-2 is recruited prior to phagosome-lysosome fusion (41). Indeed, Brefeldin A (BFA), an inhibitor of ARF GTPases that blocks protein trafficking through the early secretory pathway, inhibited perforin-2 (but not Lamp1) recruitment, consistent with the delivery of full-length perforin-2 to phagosomes from the Golgi (Fig. 4B and fig. S11, B and C). In phagoFACS experiments with the αMACPF antibody, perforin-2 acquisition was also BFA-dependent. However, the αMACPF staining was restricted to Lamp1^+^ phagosomes and increased over time, while the αC-term signal was gradually lost (Fig. 4B and fig. S11, A and B). Thus, during phagosome maturation, the C-terminal part of perforin-2 is cleaved off and degraded, while the ectodomain undergoes a conformational change exposing an epitope recognised by the αMACPF antibody.

To confirm that perforin-2 maturation in phagosomes is pH-dependent, we first asked whether MutuDC phagosomes acidify given the conflicting reports on whether the pH in DC phagosomes remains high (42–44) or decreases (45–47) over time. Using pHrodo-beads, we found that phagosome acidification in MutuDCs was abrupt and occurred on a time-scale comparable to that of Lamp1 acquisition (fig. S12). Inhibition of phagosomal acidification with BafA1 interfered with both gradual loss of the αC-term signal and acquisition of the αMACPF signal (Fig. 4B and fig. S11B), confirming that perforin-2 maturation is regulated by pH.

The pHrodo experiments also revealed that phagosomal pH was similar in NT and *Mpeg1*^KO^ MutuDC (fig. S12), suggesting that perforin-2 pores are either not permissive to protons, pore formation is transient, or the cells can compensate for proton loss. Furthermore, these data suggest that membrane integrity of phagosomes is not compromised during perforin-2-mediated escape, in line with the observation that phagosomes containing OVA-beads do not recruit galectin-3, a marker of damaged compartments (6). Consistent with these data, ovalbumin degradation was not affected by knocking out *Mpeg1* confirming that perforin-2 does not drastically alter the degradative potential of phagosomes (fig. S11, D and E).

Thus, perforin-2 undergoes pH-dependent maturation in antigen-containing compartments and perforin-2-mediated endocytic escape of antigens can occur while preserving the overall integrity of the phagosomal membrane.

## Perforin-2 is expressed in antigen-presenting cells and facilitates cross-presentation

To test whether the perforin-2 is involved in the delivery of antigens for cross-presentation, we generated *Mpeg1^-/-^* mice (fig. S13A and B). Notably, knocking out *Mpeg1* did not result in any obvious disease phenotype or a change in immune cell frequencies (Fig. S13C and D).

**Fig. 5:**
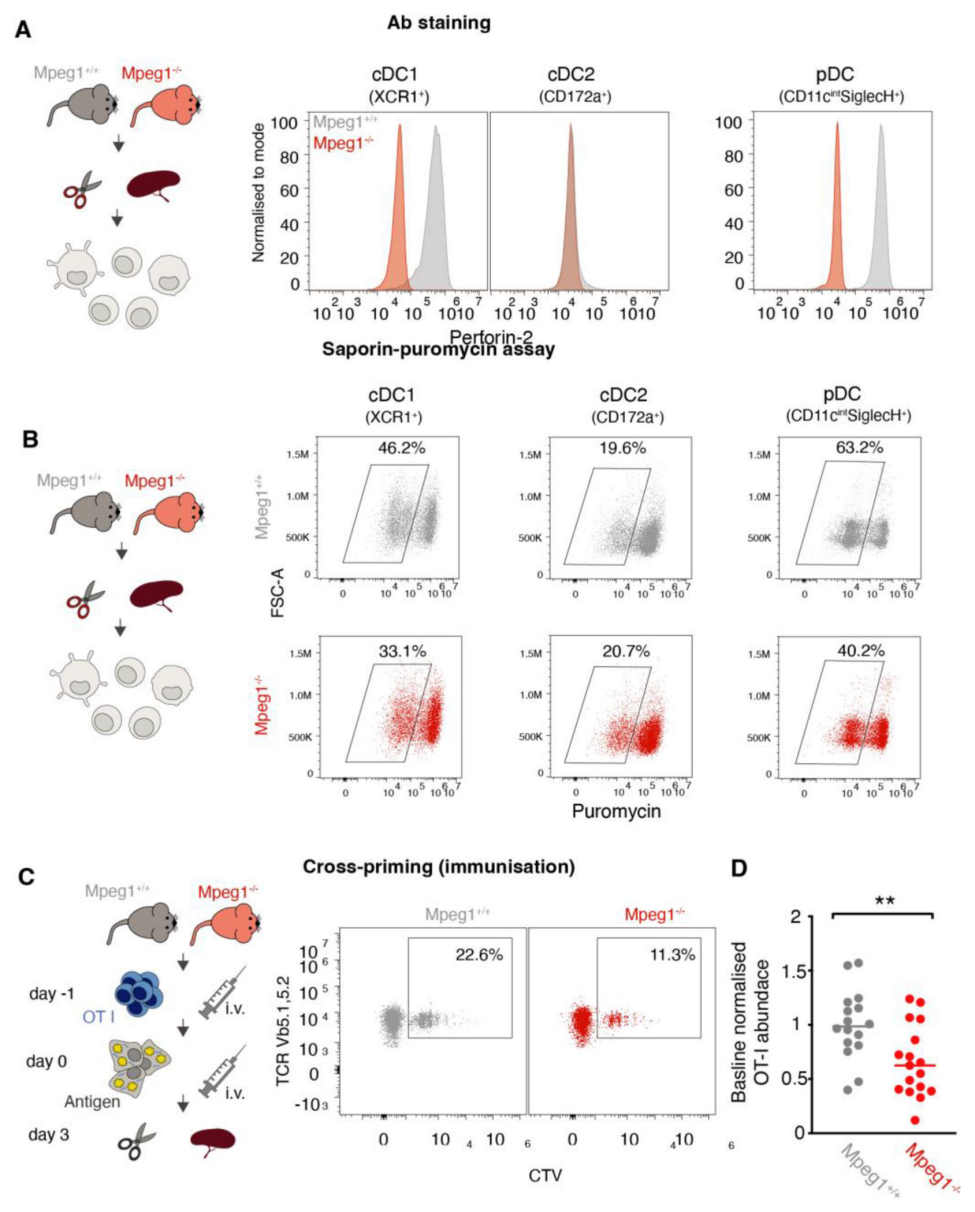
Antigen cross-priming is impaired in vivo in the absence of perforin-2. (A) Perforin-2 expression was assessed by intracellular staining with an αPerforin-2 antibody and flow cytometry in *Mpeg1^+/+^* and *Mpeg1^-/-^* splenocytes. cDC1s are defined as Lineage (CD3, CD19, NK1.1)^-^, F4/80^-^, CD11C^+^, XCR1^+^), cDC2s as Lineage (CD3, CD19, NK1.1)^-^, F4/80^-^, CD11C^+^, CD172a^+^) and pDCs as Lineage (CD3, CD19, NK1.1)^-^, F4/80^-^, CD11c^int^SiglecH^+^. For gating strategies see fig. S13C. (B) CD11c^+^ magnetically enriched splenocytes from wild-type and *Mpeg1*^-/-^ mice were pulsed with saporin for 2 h, and translation was monitored by a 30 min puromycin chase. cDC1s are defined as (CD11c^+^, XCR1^+^), cDC2s are defined as (CD11c^+^, CD172a^+^) and pDCs are defined as (CD11ci^nt^SiglecH^+^). Dot plots are representative of two independent experiments. For gating strategies see fig. S14E. (C, D) Wild-type and *Mpeg1^-/-^* mice were intravenously (i.v.) injected with 0.5x10^6^ CTV-labelled magnetically purified OT-I cells. One day later, mice were i.v. injected with 1x10^6^ UVC-irradiated (240mJ/cm^2^) 3T3 cells, coated with 10 mg/mL ovalbumin as antigen source and 0.5 mg/mL Poly(I:C) as an adjuvant. Three days later, OT-I proliferation was assessed by flow cytometry (C). OT-I are defined as Lineage (CD19, F4/80, CD11c)^-^, CD3^+^, CD4^-^CD8^+^, TCRvβ5.1, 5.2^+^TCRv**α**2^+^, CTV^+^. For gating strategy see fig. S15A. (D) Normalised OT-I counts three days after i.v. antigen injection. Each dot corresponds to an individual mouse, with three to five mice per group. For each experiment, OT-I counts per 1x10^6^ splenocytes were normalised to the average of wild-type controls. Data represent five independent experiments, ns, not significant; **P<0.01 using an unpaired t-test.

We used intracellular staining to confirm that perforin-2 expression is restricted to splenic cDC1s (Lin^-^CD11c^+^XCR1^+^), as well as other cell types previously shown to cross-present, including splenic macrophages (Lin^-^F4/80^+^), plasmacytoid DC (pDCs, Lin^-^F4/80^-^CD11c^int^SiglecH^+^), and Ly6C^+^ monocytes (Fig. 5A and fig. S14A) (48–51). In the spleen only a small, CX3CR_1_^+^ subpopulation of cDC2s expressed perforin-2, while in the lungs a large fraction of cDC2s was perforin-2 positive (fig. S14B and C). Consistent with perforin-2 expression patterns, knocking out *Mpeg1* decreased the efficiency of endocytic escape in splenic cDC1s and pDCs, but not in cDC2s (Fig. 5B).

Finally, we asked whether perforin-2-mediated escape delivers antigens for cross-presentation. To assess the efficiency of antigen presentation in vivo, we adoptively transferred OT-I T cells (expressing a TCR specific to H2-K^b^ MHC-I with ovalbumin_257-264_ peptide) and monitored OT-I proliferation following immunisation. Because cross-presentation of soluble ovalbumin, which can be processed by cell types other than cDC1s (49, 51), was not significantly affected in the Mpeg1^-/-^ mice (fig. S15), we used dead cells as an antigen source. Compared to WT mice, Mpeg1^-/-^ had fewer OT-I T cells following immunisation with UVC-irradiated, ovalbumin-coated fibroblasts (Fig. 5C). Similarly, *Mpeg1^-/-^* bone marrow-derived cDC1s (from Flt3-L and GM-CSF cultures) displayed impaired endocytic escape in the saporin assay and a reduced capacity to cross-present cell-associated antigens (fig. S16). Thus, loss of perforin-2 leads to a defect in cross-presentation of cell associated antigens in vitro and in vivo, suggesting that in cross-presenting cells, endocytic pores provide a route for cytosolic entry of antigens.

## Conclusions

Here, we uncovered a mechanism of endocytic escape that is governed by a cell type-specific pore-forming effector protein, perforin-2. The role of perforin-2 in the delivery of antigens for cross-presentation suggests that the immune system evolved to employ two related pore-forming effectors, perforin-1 and perforin-2, during different stages of adaptive immune responses. Perforin-1, expressed by cytotoxic T cells, is a well characterised effector employed for the delivery of granzymes into the cytosol of target cells (52). Our data suggest that perforin-2 delivers endocytic contents into the cytosol of cross-presenting DCs, to enable generation of MHC-I:peptide complexes and T cell priming.

We do not exclude the possibility that in different contexts, membrane destabilisation may also result in antigen delivery for cross-presentation (6, 9, 53). Membrane integrity is regulated by a wide range of mechanisms (54, 55), and different perturbations can result in leakage of endosomal contents into the cytosol under pathological conditions. However, perforin 2-mediated endocytic escape appears to occur without compromising the overall stability of the endocytic compartments.

The restricted expression of *Mpeg1* to subsets of professional antigen presenting cells, points to an important role of perforin-2 during the initiation of immune responses. The ability to genetically manipulate perforin-2-mediated endocytic escape provides a tool to explore the contribution of the escape pathway to anti-cancer and anti-viral immunity in vivo.

## Supplementary Material

Supplementary Materials

Supplementary Table 1

Supplementary Table 2

Supplementary Table 4

## Data Availability

All data are available in the main text or the supplementary materials. Data are available via ProteomeXchange with identifier PXD041861.

## One-Sentence Summary

Pore-forming protein perforin-2 promotes antigen escape from endocytic compartments in dendritic cells, enhancing cross-presentation
