# Supplementary material for: Perforin-2 is a pore-forming effector of endocytic escape in cross-presenting dendritic cells
Source: Science. Author manuscript; Available in PMC 2023 Jul 19. (PMC7614779; doi:10.1126/science.adg8802)
Supplement: Supplementary Materials [file EMS176341-supplement-Supplementary_Materials.pdf]

Science
AAAS

Supplementary Materials for

Perforin-2 is a pore-forming effector of endocytic escape in cross-presenting dendritic cells

Pablo Rodríguez-Silvestre, Marco Laub, Patrycja A. Krawczyk, Alexandra K. Davies, Julia P. Schessner, Reejuana Parveen, Benjamin J. Tuck, William A. McEwan, Georg H.H. Borner, Patrycja Kozik

Correspondence to: Patrycja Kozik pkozik@mrc-lmb.cam.ac.uk

This PDF file includes:

Materials and Methods
Supplementary Text
Figs. S1 to S16
Tables S3 and S5

Other Supplementary Materials for this manuscript include the following:

Tables S1, S2, and S4

Materials and Methods

Reagents
All reagents used in this study are listed in table S5. All antibodies used in this study are listed in table S3.

Cell Culture
MutuDCs 1940 (murine origin) were maintained in IMDM + Glutamax, 8% heat inactivated FSC (HI-FSC), 10 mM HEPES, 500 µM β-mercaptoethanol, +/- penicillin (100 units/mL)/ streptomycin (100 µg/mL). 3T3s (murine origin, CVCL_0594), HEK293Ts (human origin, CVCL_0045) and HeLas (human origin, CVCL_0030) were maintained in DMEM + 10% FCS. Isolated murine primary T cells and bone marrow cells were maintained in RPMI-1640, 10% heat inactivated FSC, 10 mM HEPES, 500 µM β-mercaptoethanol, sodium pyruvate, non-essential amino acids and penicillin (100 units/mL) / streptomycin (100 µg/mL).

For BMDC cultures, BM cells from 8–13-week-old male or female C57BL/6J mice were resuspended in media supplemented with 5 ng/mL GM-CSF and 20 ng/mL Flt3-L. 15 x 10^6 cells were seeded in a non-tissue culture treated 10 cm plate. Cells were differentiated for 9/10 days with the addition of 10 mL of fresh media with 5 ng/mL GM-CSF and 20 ng/mL Flt3-L on the fifth or sixth day and 5 mL of media containing 20 ng/mL GM-CSF added on the seventh day.

Plasmids
Plasmids used for generation of the constructs below are listed in table S4.

mMpeg1 Overexpression Construct
Mouse *Mpeg1* sgRNA-resistant DNA, was generated using GeneArt Gene Synthesis (sequence in table S4). Appropriate homology arms were added to *Mpeg1* cDNA by PCR with KOD Xtreme Hot Start DNA (primer sequences in table S4). *Mpeg1* DNA was inserted by Gibson assembly into pHR-scFv-GCN4-sfGFP-GB1-NLS-dWPRE.

mScarlet only construct
pHR-scFv-GCN4-sfGFP-GB1-NLS-dWPRE was digested with MluI-HF and NotI-HFat 37°C for 1 hour. mScarlet, with appropriate homolgy arms (primer sequences in table S4) was added by Gibson assembly.

Mpeg1^{IRES}-mScarlet construct
pHR-scFv-GCN4-sfGFP-GB1-NLS-dWPRE was digested with MluI-HF and NotI-HF at 37°C for 1 hour. For the *Mpeg1*^{IRES}mScarlet, the IRES promoter and mScarlet were added by Gibson assembly to the *m*Mpeg1 overexpression construct. The primers used to add the homology arms can be found in table S4.

BFP only construct

For the generation of the BFP-only construct, mTagBFP2 (table S4) was cloned in replacing mScarlet in the mScarlet only construct using MluI and NotI sites.

humanMpeg1^{IRES}-BFP construct

This plasmid was derived from the *Mpeg1*^{IRES}-mScarlet by replacing murine Mpeg1 for a human Mpeg1 IDT gene block (table S4) using KpnI and NotI sites. mScarlet was then replaced with ^{IRES}mTagBFP2 using MluI and NotI sites (table S4).

WT-mMpeg1restr^{IRES}-mScarlet and mMpeg1G212V/A213V^{IRES}-mScarlet and mMpeg1K251C/G286C^{IRES}-mScarlet constructs:

For this construct wildtype murine *Mpeg1* was replaced with a Twist gene fragment encoding wild-type murine *Mpeg1* with a silent point mutation at codon positions 495 to introduce a SbfI site (denoted mMPEG1restr).

mMPEG1$^{G212V/A213V-IRES}$-mScarlet and mMPEG1$^{K251C/G286C-IRES}$-mScarlet were generated by cloning IDT gBlocks harbouring the respective mutations into mMPEG1restr^{IRES}-mScarlet using the BstXI and SbfI sites. Sequence for these Twist gene fragments can be found in table S4.

Mpeg1 and AEP sgRNA constructs

The lentiBFP plasmid (a gift from A.N.J McKenzie, MRC Laboratory of Molecular Biology) was digested with BsmBI and dephosphorylated with CIP. Phosphorylated and hybridised oligos, corresponding to the sgRNA sequences (see Table S4), were ligated into the plasmid using T4 DNA ligase.

Mice

All mice were bred/maintained in pathogen-free conditions by the Medical Research Council ARES facility. Experiments were approved by the LMB Animal Welfare and Ethical Review Body and the UK Home Office. MRL/MpJ-Fas^{lpr}/J (The Jackson Laboratory, 000485) mice were a gift from L.C James (MRC Laboratory of Molecular Biology). C57BL/6, MRL/MpJ-Fas^{lpr}/J, OT-I and *Mpeg1*$^{-/-}$ (female or male) mice used were 8 to 13 weeks old at the time of the experiment.

CRISPR/Cas9 knockout mice generation

Both the crRNAs and the tracrRNA (TRACRRNA05M-5NMOL) were, ordered from Sigma-Aldrich, (HPLC-purified and 2-O-Methyl capped).

The sequence of the crRNA (functional) was GCUCAGCUUGGGGUUUACGA. Due to a design error, an additional non-functional crRNA (CGAUGAAGUGUAUACUAUUC) was injected. This crRNA has no targets in the mouse genome according to the Wellcome Sanger Institute Genome Editing tool (https://wge.stemcell.sanger.ac.uk/).

All oligos and Cas9 nuclease were initially resuspended in RNAse free 10 mM TrisHCL, 0.1 mM RNAse-free EDTA at 500 ng/mL and 200 ng/mL, respectively. Oligos and Cas9 were mixed in RNAse free 10 mM TrisHCL, 0.1 mM EDTA and incubated at room temperature for 15 min (final concentrations 20 ng/mL for both oligos and Cas9). The Cas9-gRNA complexes were injected into a C57/Ola zygote, which was then allowed to recover for 3 h prior to its transfer into a CD1 surrogate. The immunisation experiments were performed after two backcrosses, the remaining experiments after six backcrosses or where indicated, using littermate controls.

We assessed editing efficiency by Sanger sequencing of a 560 bp amplified DNA sequence from the target region (primers can be found in Supplementary Table 1). In doing so we identified three recurrent mutations in the different founder mice, which consisted of a 40-base deletion affecting CDS bases 526 to 566, a 17-base deletion spanning CDS bases 525 to 541, and lastly a 16-base deletion of CDS bases 524 to 540. Mice with the 17-base deletion were used for experiments.

Lentiviral Production

$3x10^6$ HEK293T cells were seeded per 10 cm dish. After 24 hours, cells were transfected with the sgRNA plasmid and lentiviral packing plasmids (psPAX2 and VSVG-Pmd2) using TransIT-LT1 transfection reagent. Media was changed 18 hours post-transfection to DMEM, 10% FCS, 1% BSA. Virus-containing supernatant was first collected 48 h post-transfection and media was replenished. This first virus batch was kept overnight at 4 °C. A second virus batch was collected the following day (72 h post-transfection), and both aliquots were mixed. Cellular debris was removed by centrifugation at 380 g for 5 min, before concentrating the virus by centrifugation at 3000 g for 45 min in Amicon Ultra-15 centrifugal filters.

Flow cytometry

All flow cytometry staining was done on ice. For cell surface staining, nonspecific binding was blocked using αCD16/CD32. Cells were then stained with the corresponding antibodies for 30-60 min (table S3). Following antibody staining, cells were washed three times with PBS, 1mM EDTA, 1% FCS. For live/dead staining cells were either resuspended in a (0.1 μg/mL) DAPI PBS, 1mM EDTA, 1% FCS solution or stained for 10 min with a fixable viability dye (table S3). Intracellular staining was done using BD Cytofix/Cytoperm Fixation/Permeabilization Kit. Data was acquired on a LSRFortessa (BD) or a CytoFLEX (Beckman Coulter) and analysed on FlowJo 10 (BD). For cell sorting, cells were sorted into serum coated polypropylene tubes using a SY3200 Cell Sorter (Sony).

Western Blotting

Cell pellets were lysed in RIPA buffer with protease inhibitor for 20 min at 4°C while shaking at 800 rpm. Lysates were centrifuged at 20,000g for 10 min at 4°C, the supernatant was then transferred to a clean 1.5 mL Eppendorf. Lysates were mixed with LDS NuPAGE + Bolt reducing agent and incubated at 70°C for 10 min. Samples were loaded onto an SDS-Page gel and transferred onto a nitrocellulose membrane using an iBlot1 (Invitrogen). The membrane was blocked for 1 h with 5% milk solution in PBS, 0.01% Tween (PBST), and blotted with the primary antibody overnight at 4°C. Secondary staining was performed for 1h at RT. Chemiluminescence was detected with ECL Prime Western Blotting Detection Reagent.

Saporin assay

3 x 10^5 cells were seeded per well in a tissue culture treated 96-well U-bottom plate. Cells were pulsed for 2 h at 37°C with saporin (Merck, S9896). Cells were spun at 380g for 3 min and resuspended in DC media containing 0.01 mg/mL puromycin and incubated for 30 min at 37°C.

To monitor saporin-mediated activation, 3 x 10^5 cells were seeded per well in a tissue culture treated 96-well U-bottom plate. Cells were pulsed with saporin for 3 h at 37°C degree. Cells were washed once in PBS, stained and analysed by flow cytometry.

For the CRISPR/Cas9 screen, 3 x 10^6 cells were seeded per well in a tissue culture treated 6-well plate. Cells were pulsed with 0.5 mg/mL saporin for 2 h at 37°C. Cells were then washed twice with media and incubated with puromycin at 0.01 mg/mL in DC media for 30 min at 37°C.

Saporin was labelled with an ATTO 550 protein labelling kit (Merck, 51146-1KT).

Bead saporin

Bead preparation

Saporin or BSA were conjugated to Ova-beads through disulfide bonds. For efficient conjugation, free sulfhydryl groups were introduced by reacting 0.6 mL of 2.5 mg/mL saporin or BSA with 12 µL of 2 mg/mL Traut's reagent in PBS + 2 mM EDTA for 60 min at room temperature. Excess reagent was removed using a Zeba spin desalting column equilibrated with PBS + 2 mM EDTA.

Ova-beads were resuspended in PBS + 2 mM EDTA and reacted with 1 mM SPDP for 30 min at room temperature. The beads were then washed twice in PBS + 2 mM EDTA and incubated with 1 mg/mL cysteine-modified saporin or BSA overnight at room temperature. After incubation, beads were washed twice in PBS and immediately used for phagocytosis.

Bead-based saporin assay

The assay was performed in cell culture medium lacking β-mercaptoethanol. MutuDCs were collected, washed once in PBS and seeded in a 96-well U-bottom plate with 5 x 10^5 cells per well in 100 µL cell culture medium. Saporin/Ova- or BSA/Ova-beads were diluted in cell culture medium such that adding 50 µL of each dilution to cells gave a 10:1 ratio of beads:cells. At the end of each incubation, puromycin was added at 0.01 mg/mL for 30 min and cells were washed in ice-cold PBS. Non-internalised beads were labelled in PBS containing 1% (vol/vol) BSA with a rabbit αOvalbumin antibody for 30min on ice followed by donkey αRabbit-AF555 for 30min on ice. After labelling dead cells with a fixable viability stain for 10 min on ice, cells were fixed and permeabilised using the BD Fix/Perm and Perm/Wash buffers. Puromycin incorporation was determined by staining with an αPuromycin-AF647 antibody in Perm/Wash buffer for 45 min on ice. Cells were analysed by flow cytometry.

β-lactamase assay

4 x 10^6 HeLa cells were pulsed with 800 uL of the CCF4 solution (prepared as in (*19*) for 45 min, and washed by adding 5 ml PBS and spinning at 450 g, 15°C for 5 min. The cells were there resuspended in warm HeLa media with Probenecid at a 1:100 dilution (ThermoFished, P36400). To control for the background conversion of CCF4 observed in the absence of β-lactamase, each sample was split into two, media-only or media with β-lactamase (final concentration 2 mg/mL; P0389, Sigma). At each time point, 100 uL of the cell suspension was transferred into 100 uL of ice-cold PBS to stop trafficking. Finally, the cells were centrifuged for 2 min at 800 g, 4°C, stained with eFluor 780 live/dead solution containing Probenecid for 10 min on ice, centrifuged again, and resuspended in FACS buffer with Probenecid for flow cytometry.

MutuDC survival assays

For saporin, gelonin and cycloheximide treatments, 5 x 10^4 cells were seeded per well in a treated 96-well U-bottom plate. Cells were incubated with the different treatments for 24 hours. Cells were washed twice in PBS and then stained with a fixable viability stain. Cell viability was assessed by flow cytometry.

For treatment with Poly (I:C) 2.5 x 10^4 cells were seeded per well in a treated 96-well U-bottom plate. Poly (I:C) was added and cells were incubated for 48 hours. Cells were washed in twice in PBS and then stained with a fixable viability stain. Cell viability was assessed by flow cytometry.

For treatment with Bleomycin A1, 2 x 10^5 cells were seeded per well in a treated 96-well U-bottom plate. Bleomycin A1 was added and cells were incubated for 48 hours in the Incucyte®. The increase in cell area covered by the cells was monitored for each well and normalised to untreated cells.

TLR treatments

1.5 x 10^6 cells were plated in a tissue culture treated 6 well plate and allowed to adhere for 7 h. MutuDCs were then pulsed with the indicated TLR ligands for 16 h at 37°C. TLR ligands were used at the following concentrations: PAM3CSK4 (1 μg/ml), FSL-1 (1 μg/mL), HMW Poly(I:C) (5 μg/mL), CRX-527 (1 μg/mL), LPS (5 μg/mL), flagellin (100 ng/mL), ssPoly(U) (10 μg/mL), R848 (10 μg/mL), CpG ODN 2395 (1 μM), profilin (1 μg/mL), ORN sa19 (2 μg/mL). MutuDCs were collected and prepared for analysis by Western blot.

PhagoFACS

Bead preparation

To generate Ova-beads, amino-modified microspheres with a diameter of 3-μm were washed twice in PBS and preactivated with 8% (vol/vol) glutaraldehyde for 4 h at room temperature. Preactivated beads were washed once in PBS and then incubated overnight at 4°C with ovalbumin at a concentration of 0.5 mg/mL in PBS. After incubation, beads were quenched in 0.4 M glycine in PBS, washed twice in PBS and used immediately for phagocytosis.

PhagoFACS assay

MutuDCs were collected, washed once in PBS and resuspended in ice-cold internalisation medium (CO2-independent medium containing 1X GlutaMAX) to a density of 20 x 10^6 cells/mL. Ova-beads were added at a 10:1 ratio of beads:cells and incubated for 25 min at 16°C followed by a 5min incubation at 37°C to allow phagocytic binding and internalisation of beads. To remove non-internalised beads, cells were first washed twice with 10 mL ice-cold PBS at 100 g for 4 min at 4°C and then resuspended in 1 mL PBS, applied to a 5 mL FCS cushion and centrifuged at 150 g for 4 min at 4°C. The cell pellet was then resuspended to 20 x 10^6 cells/mL in cell culture medium (containing 5 µM BFA or 0.1 µM BafA1 for drug treated cells) and divided into different time points comprising 5 x 10^6 cells each. The chase was performed at 37°C for different periods of time and stopped by adding ice-cold PBS. Non-internalised beads were labelled by staining with a goat αOvalbumin antibody for 30 min on ice followed by an αGoat-AF488 antibody for 30min on ice. Cells from each time point were resuspended in 0.5 mL homogenization buffer (250 mM sucrose, 3 mM imidazole, 2 mM DTT, 2 mM PMSF and 1X protease inhibitor cocktail, ph 7.4) and passed 25 times through a 22-G needle. Intact cells and debris were pelleted by centrifugation at 150 g for 4min and the phagosome-containing post-nuclear supernatants transferred to a V-bottom 96-well plate. The enriched phagosomes were washed with PBS containing 1% (vol/vol) BSA and stained with different primary antibodies overnight at 4 °C. The next day, the samples were incubated with appropriate secondary antibodies for 45 min on ice. Phagosomes were analysed by flow cytometry.

pHrodo assay

To generate pHrodo beads, amino-modified microspheres with a diameter of 3-µm were washed twice in PBS, resuspended in 100 mM sodium bicarbonate (pH 8.5) and reacted with 0.2 mM pHrodo iFL Red Ester dye for 1 h at room temperature. The beads were then washed once in PBS and any reactive ester moieties were quenched by incubating the beads in 1X TBS for 10 min. After an additional two washes in PBS, the beads were coated with 1 mg/mL ovalbumin by passive absorption for 1 h at room temperature. The beads were then washed twice in PBS and immediately used for phagocytosis as described for the phagoFACS assay. Non-internalised beads and dead cells were labelled by staining with a rabbit αOvalbumin antibody in PBS containing 1% (vol/vol) BSA for 30 min on ice followed by a staining with a donkey αRabbit-AF647 antibody and ViaKrome 808 in PBS for 30 min on ice. Stained cells were immediately analysed by flow cytometry using a chilled sample stage.

Microscopy

Immunofluorescence, galectin 3 recruitment

1 x 10^5 MutuDCs were plated on a µ-slide 8 well dish and allowed to adhere at 37°C overnight before treating them with 33 µM GPN for 10 min at 37°C. Cells were washed three times in PBS and fixed in 4% paraformaldehyde for 10 min at RT. Paraformaldehyde was washed away before permeating cells with 0.1% Triton-X100 for 10 min at RT. Cells were washed 3 times in PBS, and incubated for 30 min at RT with blocking buffer (1% BSA, 0.3M glycine, 0.1% Tween 20 in PBS). After three PBS washes, cells were stained with αGalectin3 for 40 min and then washed 3 times

in PBS. Cells were then stained with donkey αMouse-Af647 and then washed three times in PBS. Images were acquired on a Zeiss 780 inverted confocal microscope. The images were processed and analysed in Fiji.

Immunofluorescence, co-localisation with perforin-2

1.5 x 10^5 MutuDCs were plated on poly-L-lysine coated μ-slide 8 well dish and allowed to adhere at 37°C overnight. To label acidic compartments, the cell culture medium was replaced with fresh medium containing 1 μM LysoTracker Red and cells were incubated for 30 min at 37°C. Cells were then washed twice with PBS and fixed in 4% formaldehyde for 10 min at RT. After two washes with PBS, the samples were permeabilised with 0.15% Triton X-100 in PBS for 10 min at RT followed by three washes with PBS. To block unspecific binding and quench excess formaldehyde, the cells were incubated in blocking buffer (1% BSA, 0.3M glycine, 0.1% Tween 20 in PBS) for 30 min. Primary and secondary antibody incubations were performed in PBS containing 1% BSA for 1 hr at room temperature with three washes in PBS after each incubation. Nuclei were labelled by incubating the cells for 10 min with DAPI followed by two washes in PBS. Images were acquired on a VisiTech iSIM swept field confocal super resolution system coupled to a Nikon Ti2 inverted microscope stand equipped with a 100x/1.49 NA SR Apo TIRF objective lens. The images were processed and analysed in Fiji.

Tau entry assay

Tau entry assays were performed as previously described (*36*). Briefly, HEK 239T cells expressing NLS-eGFP-LgBiT (NGL) were transduced with lentivirus harbouring human*Mpeg1*^{IRES}BFP or BFP only under the control of an SFFV promoter. Approximately 16 h prior to assay, 2x10^4 cells were seeded into a white 96-well plate coated with poly-L-lysine. The media was replaced 16 h later with recombinant 0N4R-P301S-tau-HiBiT (tau-HiBiT) protein at desired concentration in Assay Medium composed of CO2 independent medium supplemented with 1% penicillin-streptomycin, 1 mM GlutaMAX and 1 mM sodium pyruvate. Cells were washed once with PBS, and incubated in substrate solution (Assay Medium, LCS buffer and Live Cell Substrate) at RT for 5 min before loading onto a pre-warmed clarioSTAR Microplate Reader at 37ºC. The plate was mixed by 200 RPM double orbital shaking for 10 seconds prior to signal acquisition by spiral average (NanoLuc setting, 470 nM). Post-signal acquisition, cell viability per well was acquired by incubation with PrestoBlue Viability Reagent according to manufacturer instructions. The plate was then loaded onto the ClarioSTAR Microplate Reader and fluorescence read by excitation wavelength of 560 nm and emission at 590 nm.

Processing of mouse tissues

Spleens were perfused with a solution of RPMI-1640, 0.1 mg/mL Liberase-TL and 0.1 mg/mL DNAse I, minced and digested for 25 min at 37 ˚C. HI-FCS was added (10% v/v) to stop the digestion, before filtering tissues through a 70 μM filter. Red blood cells were then lysed with red blood cell lysis buffer hybrid-max for 3 min at RT. Lungs were minced in a solution of RPMI-1640, 750 U/mL collagenase type I and 0.3 mg/mL DNAse I. Samples were digested for 30 min at 37 ˚C while shaking at 800 rpm. Digest was then filtered through a 70 μM filter before washing

twice with PBS 2% HI-FCS. Red blood cells were then lysed in 140 mM NH_4Cl, 17mM Tris, pH 7.2 for 5 min at RT. To obtain the bone marrow, femurs and tibias from mice were cut at both ends, and the bone marrow was flushed into BMDC media by brief centrifugation at 10,000g. Red blood cells were lysed with red blood cell lysis buffer hybrid-max for 1 min at RT. Tissue CD11c+ cells were enriched using a Pan-Dendritic Cell Isolation Kit. OT-I T cells were obtained from OT-I spleens and lymph nodes. Both tissues were mashed and filtered through a 70 µM filter. OT-I T cells were enriched using either EasySep Mouse Naïve CD8+ T Cell Isolation Kit or Naïve CD8a+ T cell isolation kit.

Cross-priming assays

3T3 UVC-irradiation and antigen coating

1.5 x 10^6 3T3 cells were plated in 10 cm plate 16 hours prior to irradiation. For irradiation, media was replaced with 5 mL PBS. Cells were UVC irradiated (240 mJ/cm^2) with a UVP Crosslinker (AnalytikJena). Media was then replenished, and cells were incubated for 16 h at 37°C.

For antigen coating, the supernatant, a PBS wash and the trypsinised UVC-irradiated 3T3s were collected and resuspended at 10 x 10^6 cells/mL in 10mg/mL ovalbumin and 0.25 mg/mL HMW Poly(I:C). Cells were incubated for 1 h at 37°C. Cells were washed three/four times in ice-cold PBS and resuspended for injections.

OT-I CTV labelling

Staining was performed with a CellTrace Violet Cell Proliferation Kit. Isolated cells were resuspended at 5 x 10^5/mL in a 2.5 µM CellTrace Violet solution, and incubated at 37°C for 20 min in the dark. 10% v/v FSC was added, and cells were incubated a further 5 min at 37°C. Cells were centrifuged at 300 g for 5 min, resuspended in T cell media and incubated at 37°C for 10 min.

In vivo immunisation

8–12 week old male and female mice were i.v. injected 0.5 x 10^6 OT-I cells. The following day mice were injected with either 100 µg of ovalbumin + 50 µg Poly(I:C), or with 1x10^6 UVC-irradiated 3T3s coated as described with 10 mg/mL ovalbumin and 0.5 mg/mL Poly(I:C). Three days later spleens were isolated and OT-I T cell abundance was assessed by flow cytometry.

In vitro cross-presentation assay

$XCR1^+$ cDC1s were magnetically enriched from day 9 Fl3L-GM/CSF cultures using an EasySep PE Positive Selection Kit and XCR1-PE antibody (2 µg/mL). Isolated cDC1s were then plated at 1 x 10^5 cells per well in a U-bottom 96 well plate. UVC irradiated 3T3s coated were added to cDC1s and incubated for 20 h at 37 °C. Cells were then washed twice in PBS and 5 x 10^4 CTV-labelled OT-Is were added and co-cultured for 3 days at 37 °C. OT-I proliferation was then assessed by flow cytometry.

Recombinant perforin-2 cleavage

Expression and purification of recombinant perforin-2

The murine perforin-2 ectodomain (amino acids 20-652), tagged with an N-terminal signal peptide and a C-terminal hexahistidine tag was introduced into vector pHL-sec. Expi293F cells at $2x10^6$/mL were transfected with 3.8 mg of plasmid in the presence of 10.2 mg of Polyethyleneimine Max. Three to four hours later, valporic acid was added at a final concentration of 3.5 mM. Cells were allowed to grow for a further 5 days. Culture media was collected, centrifuged for 2 h at 4000g and filtered through a 0.22 µM membrane. An ÄKTA flux^{TM} (Cytiva Life Sciences) was used for sample concentration (with a 10kDa cut-off filter) and buffer exchange to Buffer A (25mM Tris pH 7.5, 500Mm NaCL, 10 mM imidazole). The sample was then loaded onto a Ni-NTA column at RT. The column was washed, sequentially, with 10 mM and 40 mM imidazole, before collecting the perforin-2 elute with a 200 mM imidazole wash. The perforin-2 fractions were then dialyzed in PBS and concentrated to 2 mg/mL.

Asparagine Endopeptidase cleavage reactions

AEP (specific activity 350 pmol/min/µg) was resuspended in activation buffer (0.1 M NaOAc, 0.1 M NaCl, pH 4.5) at 50 µg/mL. Prior to the cleavage reactions, AEP was incubated for 4 h at 37°C. For the *in vitro* cleavage reactions, AEP was diluted in assay buffer (50 mM MES, 250 mM NaCl, adjusted pH 5.5) and 35 or 175 µU were added to a 50 µL final reaction volume. To ensure AEP was active, cleavage of a fluorogenic substrate was confirmed using manufacturer's protocols. For perforin-2 cleavage, 4 µL of purified perforin-2 was cleaved in the absence or presence of AEP inhibitor peptide at 0.5 mg/mL. Reactions were allowed to proceed for 2 h at 37°C before being terminated by addition of denaturing agent.

CRISPR/Cas9 Screen

sgRNA library design

Microarray expression data for splenic cDC1s ($CD8\alpha^+$ DCs) and cDC2s ($CD4^+$ DCs) was downloaded from Immgen (www.Immgen.org) (*35*) on Feb 5th 2017. Genes with log_2(cDC1/cDC2) expression ratio > 1.3 were selected for the minilibrary (table S1). Four sgRNA sequences per gene were picked from the genome-wide Brie library (*36*). For genes not present in the Brie library, the sgRNAs were designed using the Broad Institute Genetic Perturbation Platform portal: https://portals.broadinstitute.org/gpp/public/analysis-tools/sgrna-design. The resulting library targeted 281 genes (table S1).

sgRNA library generation

The oligos to generate the sgRNA library were ordered from Twist Bioscience as part of a larger pool containing several libraries. The sequences are listed in table S1. The oligo pool was resuspended to 53 nM and minilib-PCR1 primers (table S1) were used to amplify the library.

The LentiBFP vector was digested with BsmBI, and a gibson reaction was set up with the library inserts. The Gibson reaction mix was electroporated into EnduraTM competent cells and

electroporated at 1.8 kV/600O/10µF. Bacteria were allowed to recover in recovery media for 1 h at 37°C, before plating them in a Luria broth (LB) lennox NuncTm Square BioAssay dish and growing them overnight at 30°C. Bacteria were then collected by washing the plate 5 times with 5 mL LB. Bacteria were centrifuged at 4000 g for 15 min, before removing the supernatant and freezing the pellet. For DNA purification, the bacteria pellet was thawed, and DNA purified using a QIAGEN Plasmid Maxi Kit.

Lentiviral Transduction for the CRISPR-Cas9 screen

Cas9 expressing MutuDCs were seeded at $2x10^6$ per 10 cm dish. Virus was added at a 0.3 MOI. Two days after transduction BFP positive cells were sorted and allowed to recover. Library representation was maintained at 800x.

Preparation of libraries for next generation sequencing

Genomic DNA was isolated using a DNeasy Blood and Tissue Kit. Samples were digested for 4 hours with Proteinase K. sgRNAs were then amplified in a two-step PCR with Herculase II Fusion DNA Polymerase. For the first PCR a maximum of 5 ug of DNA per 50 µL reaction was amplified using all available genomic DNA and libgen-PCR1 primers (table S1). The reaction mixed consisted of 10 µL 5X Herculase II reaction buffer, 0.5 µL dNTP mix, 0.5 µL Herculase II fusion DNA polymerase and 1.25 mL of 10 µM both the forward and reverse primers. PCR cycling conditions were: 1 min at 95 °C; 18x (30 s at 95°C, 30 s at 55 °C, and 30 s 72 °C); 10 min at 72°C).

The number of cycles for the second PCR was determined by qPCR using a KAPA Library Quantification Kit. Cycling conditions were: 2 min at 95 °C, followed by 30 s at 95 °C, 30 s at 53 °C and 30 s at 72 °C for a total of 40 cycles. Samples with similar CT values were then pooled for the second PCR.

Following sgRNA amplification from gDNA, a second PCR was performed to barcode the sgRNAs using 10 µL from the first PCR. PCR cycling conditions were: 2 minutes at 95 ˚C, followed by 30 s 95 °C, 30 s at 53 °C and 30 s at 72 °C for the number of cycles determined by the previous qPCR step, and a final 10 min at 72 °C. The amplified products were separated using a 2% agarose gel and purified using a Gel Extraction Kit. Agarose was melted at 40 °C instead of the recommended 50 °C. Samples were then further purified using a Charge Switch PCR Clean-Up Kit. Finally, the concentration of DNA fragments containing P5 and P7 adaptors was determined via qPCR using KAPA Library Quantification Kit. This ensured adequate cluster density during NSG. This was done using a KAPA Library Quantification Kit. Samples were analysed by SE50 sequencing on a HiSeq4000 sequencer loaded with 15% spike-in PhiX Control Library (Illumina).

Analysis of the CRISPR-Cas9 screening data

Processing of the sequencing data

The analysis pipeline was adapted from (*56*). Demultiplexing was performed using the demuxFQ package. Next, the fastq files were processed using cutadapt-1.4.1 to remove the flanking

sequences: GACGAAACACCG and GTTTTAGA on the 5' and 3' end respectively (analysis parameters: -e 0.2 --minimum-length 20 --discard-untrimmed). The trimmed 20 bp long reads were matched to the library of reference sgRNA sequences and counted (analysis parameters: -f -v 0 -m 1 --norc -a --best --strata --un). Raw counts for each sgRNA are provided in table S1.

Hit calling

Each screen repeat was analysed initially using a modified stat.wilcox function from the caRpools (v 0.83) package with the following parameters and modifications. Guides with less than 20 counts in either of the populations selected for comparison were excluded from the analysis. The counts were then normalized to median of the population. The function returns enrichment score for each population of four sgRNAs targeting one gene relative to 100 random guides. The p-values are calculated using a two-sided Mann-Whitney-U test and non-adjusted P-values were used in the next step of the analysis.

To combine the data from the biological replicates of the screen, for each gene we calculated the mean enrichment score and used Fisher's method to combine the p-values. P-values were then adjusted using the Benjamini-Hochberg method. Genes with enrichment scores greater than 0.5 and adj p-values < 0.01 were considered as hits.

Analysis of organellar mapping data

Peptide data from organellar mapping experiments (*19*) in control, prazosin- and tamoxifen-treated cells were included in the analysis. As in the original paper, SILAC ratios were inverted, weighted with fraction yields, and divided by the sum of all five ratios across the map. This yielded for each peptide a 'per map' normalized profile (summing to 1). Mean profiles from each treatment group were used to prepare the map using the prcomp function in R.

Whole cell lysate proteomic analysis

Cell culture and treatments

$10x10^6$ MutuDCs were plated in a 90mm tissue culture treated plate and either CpG- (1 μM), BafA1-(1 μM) or mock treated for 16 h at 37 °C. Cells were then harvested with PBS 5mM EDTA, and washed three times in PBS.

Cell lysis and in-solution digestion of proteins

Cell pellets were thawed, lysed in 200 µl 2.5% (w/v) SDS/50mM Tris pH 8 and incubated at 72°C for 5min. Lysates were then sonicated at 4 °C (three times 5s bursts with an amplitude of 10µm) to break-up DNA. Estimations of protein concentrations were made using a Pierce BCA Protein Assay Kit. For each sample, 100 µg protein was precipitated by the addition of 5 volumes of ice-cold acetone, incubated at –20°C overnight and pelleted by centrifugation at 4°C for 5min at 10,000×g. All subsequent steps were performed at room temperature. Precipitated protein pellets were air-dried for 5min, resuspended in 50 µl digestion buffer (50mM Tris pH 8.1, 8M Urea, 1mM DTT) and incubated for 20min. Protein was alkylated by addition of 5mM iodoacetamide for 20min (in the dark) and then enzymatically digested by addition of LysC (1mg per 50mg of protein) for an overnight incubation. Digests were then diluted four-fold with 50mM

Tris pH 8.1 before addition of Trypsin (1mg per 50mg of protein) for 4 hours. The peptide mixtures were then acidified to 1% (v/v) TFA in preparation for peptide purification and fractionation.

Peptide purification and fractionation

For each sample, 20 µg peptides were loaded onto an SDB-RPS StageTip for peptide cleanup and triple fractionation as previously described (*57*). StageTips were activated by washing with 100 µl acetonitrile, followed by 100 µl 30% (v/v) methanol, 1% (v/v) TFA, and then 100 µl 0.2% (v/v) TFA. Peptide mixtures in 1% TFA were loaded onto activated StageTips and washed with 100 µl isopropanol and then 100 µl 0.2% (v/v) TFA. Peptides were then eluted successively using 20µL SDB-RPSx1 (100mM ammonium formate, 40% (v/v) acetonitrile, 0.5% (v/v) formic acid), then 20µL SDB-RPSx2 (150mM ammonium formate, 60% (v/v) acetonitrile, 0.5% formic acid), then 30µL SDB-RPSx3 (80% (v/v) acetonitrile, 5% (v/v) ammonium hydroxide). Peptides were dried in a centrifugal vacuum concentrator, resuspended in 10µL Buffer A* (0.1% (v/v) TFA, 2% (v/v) acetonitrile) and stored at −20°C until analysis by mass spectrometry.

Mass spectrometry

For proteomic analysis of BafA1 and CpG induced changes in the abundance of perforin-2 peptides, 500 ng of peptides were loaded on a 50 cm by 75 µm inner diameter column, packed in-house with 1.8 µm C18 particles (Dr Maisch GmbH, Germany). Peptide separation by reverse phase chromatography was performed using an EASY-nLC 1000 (Thermo Fisher Scientific), running a linear gradient over 95 min at 300 nl/min flow rate and 55 °C. The gradient ran from buffer A (0.1% (v/v) formic acid) containing 5% buffer B (80% (v/v) acetonitrile, 0.1% (v/v) formic acid) to buffer A containing 30% buffer B. Runs were separated by 5 min wash-outs with 95% buffer B and re-equilibration. The LC was coupled to a Q Exactive HF-X Hybrid Quadrupole-Orbitrap mass spectrometer via a nanoelectrospray source (Thermo Fisher Scientific). MS data were acquired using a data-dependent top-15 method that dynamically excludes precursors picked during the last 30 seconds. MS1 survey scans were acquired at a resolution of 60,000 in a 300-1650 Th range. The maximum injection time was 20 ms for up to 3e6 target ions, as determined with predictive automatic gain control. Sequencing was performed via higher energy collisional dissociation fragmentation of ions isolated from a 1.4 Th window. The maximum injection time was 28 ms for 1e5 target ions. MS2 fragment scans were acquired at a resolution of 15,000 in a 200-200,000 Th range.

For proteomic analysis of perforin-2 peptides in AEP knockout cells, 300 ng of peptides were loaded on a 50 cm by 75 µm inner diameter column, as above. Peptide separation by reverse phase chromatography was performed using an EASY-nLC 1200 (Thermo Fisher Scientific), running a linear gradient over 100 min at 300 nl/min flow rate and 50 °C. The gradient ran from buffer A (0.1% (v/v) formic acid) containing 5% buffer B (80% (v/v) acetonitrile, 0.1% (v/v) formic acid), to buffer A containing 30% buffer B in 84 min, followed by an increase to 60% buffer B in 8 min, a further increase to 95% buffer B in 4 min, and a constant phase at 95% buffer B for 4 min. Runs were separated by 5 min wash-outs with 95% buffer B and re-equilibration. The LC was coupled to an Orbitrap Exploris 480 mass spectrometer via a nanoelectrospray source (Thermo Fisher Scientific). MS data were acquired using a data-dependent top-15 method as described above. The maximum injection time for MS1 survey scans was 25 ms for up to 3e6

target ions. MS2 fragment scans were acquired at a resolution of 15,000 with a scan range starting from 100 Th.

Processing of mass spectrometry data

Mass spectrometry raw files were processed in MaxQuant Version 1.6.10.43 (*58*), using the mouse SwissProt canonical and isoform protein database, retrieved from UniProt (2019_10_22; www.uniprot.org). Label-free quantification was performed using the MaxLFQ algorithm (*59*). No matching between runs was used. To detect peptide fragments resulting from other cleavage events than the in-solution digest with LysC and trypsin, the enzyme mode was set to semi-specific. LFQ minimum ratio count was set to 1 and default parameters were used for all other settings.

To assess abundance of peptides independent of protein abundance changes, peptide intensities were divided by their corresponding protein intensities. Data were filtered for 3 valid values in at least one experimental condition and then subjected to a two-sided student's t-test. Multiple hypothesis correction was done by permutation based FDR with s0=0.1 using Perseus (*60*).

The mass spectrometry proteomics data have been deposited to the ProteomeXchange Consortium [http://proteomecentral.proteomexchange.org] via the PRIDE partner repository with the dataset identified PXD041861.

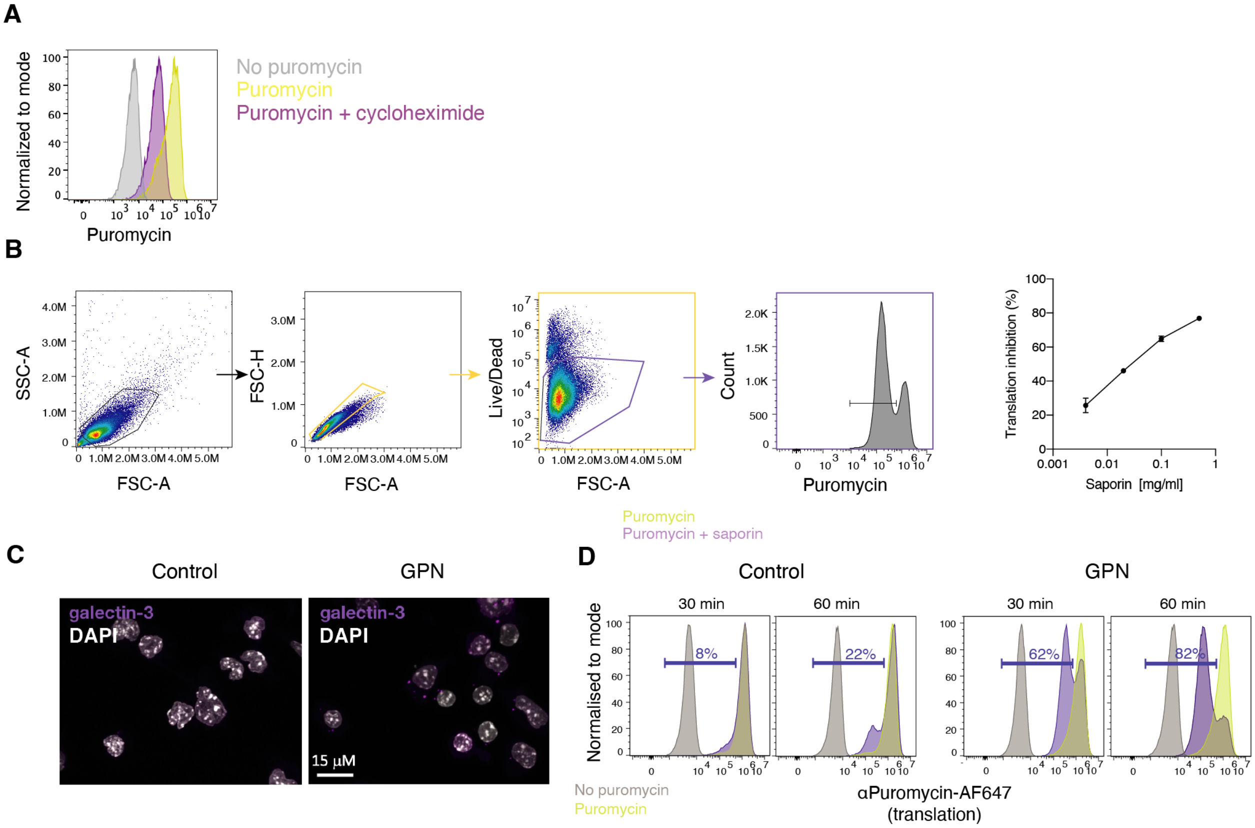

Fig S1. Saporin-puromycin escape assay in MutuDCs.

(A) Puromycylation in MutuDCs is sensitive to changes in the rate of translation. MutuDCs were incubated for 2 h at 37°C with 10 µg/mL cycloheximide followed by a 30 min 0.01 mg/mL puromycin chase in the presence or absence of 10 µg/mL cycloheximide. Puromycin incorporation was monitored with a αPuromycin antibody. Histograms are representative for three independent experiments.

(B) Saporin-induced translational arrest is dose-dependent. Flow cytometry gating strategy to monitor saporin-mediated translation inhibition in MutuDCs.

(C, D) GPN facilitates endosomal escape of saporin. To demonstrate that endocytic escape of saporin is the rate-limiting step in the saporin-puromycin assay, we disrupted the integrity of endocytic compartments with glycyl-L- phenylalanine 2-naphthylamide (GPN). GPN is a cathepsin C substrate that induces osmotic lysis of lysosomes leading to release of lysosomal contents (*61*). (C) Confocal microscopy images of MutuDCs treated with 33 µM GPN for 10 min. Cells were stained with DAPI (white) and for galectin-3 (magenta). (D) MutuDCs were incubated with 0.5 mg/mL saporin for the indicated time in the presence or absence of 33 µM GPN. Following a 30 min puromycin chase, translation inhibition was monitored by flow cytometry. Data represent three independent experiments.

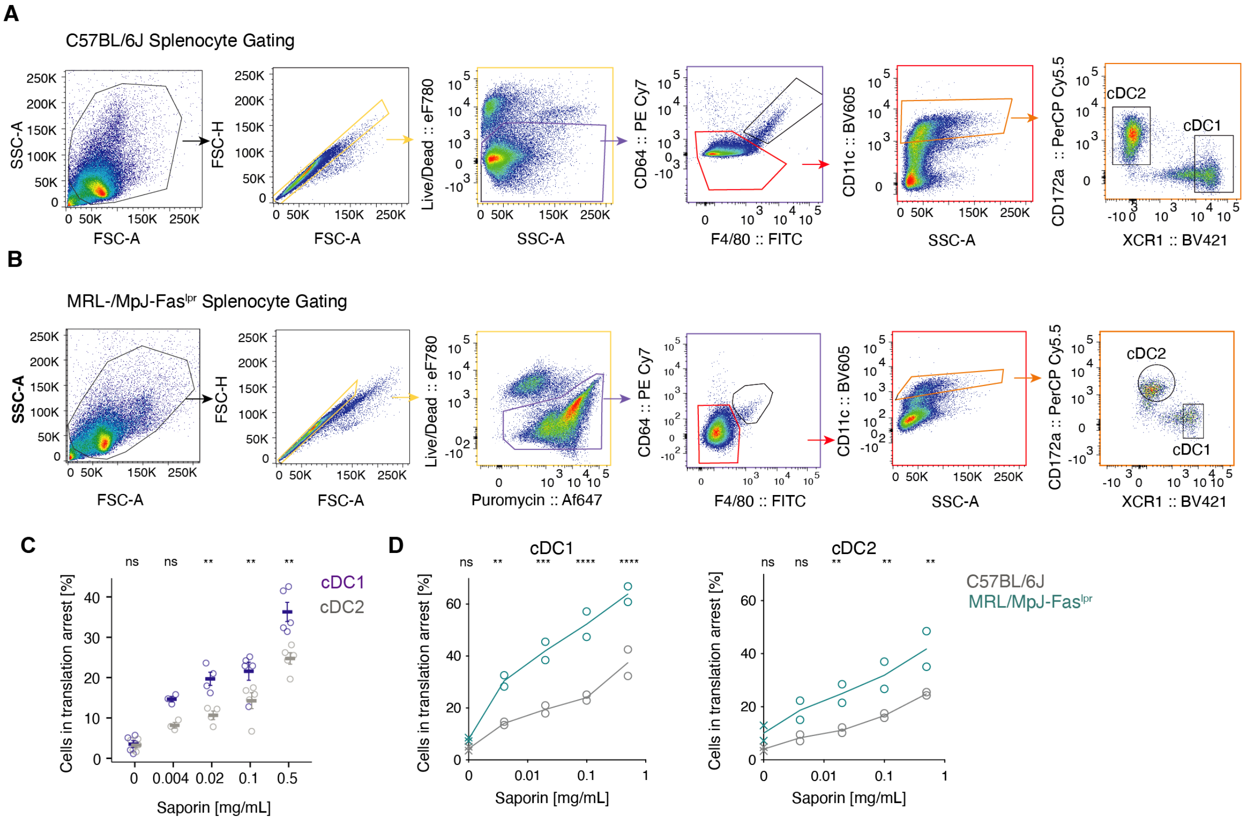

Fig S2. Saporin-puromycin assay can capture physiological differences in endocytic escape.

(A, C) CD11c+ enriched splenic DCs from C57BL/6J were incubated with saporin for 2 h, followed by a 30 min puromycin chase. Translation inhibition was monitored by flow cytometry. (A) Gating strategy for C57BL/6J splenic cDC1s and cDC2s for the *ex vivo* saporin assay. (C) Data represent mean and SEM for five independent experiments, ns, not significant; **, P<0.01 using a multiple unpaired t-test (two-stage step-up, Benjamini, Krieger and Yekutieli).

(B, D) CD11c^{+} enriched splenic DCs from C57BL/6J or MRL/MpJ-Fas^{lpr} were incubated with saporin for 2 h, followed by a 30 min puromycin chase. Translation inhibition was monitored by flow cytometry. (B) Gating strategy for MRL/MpJ-Fas^{lpr} splenic cDC1s and cDC2s for the *ex vivo* saporin assay. (D) Data from two independent experiments each with two technical replicates, ns, not significant; *P<0.5; ** P<0.01; *** P<0.001; ****P<0.0001 using a multiple unpaired t-test (alpha = 0.5).

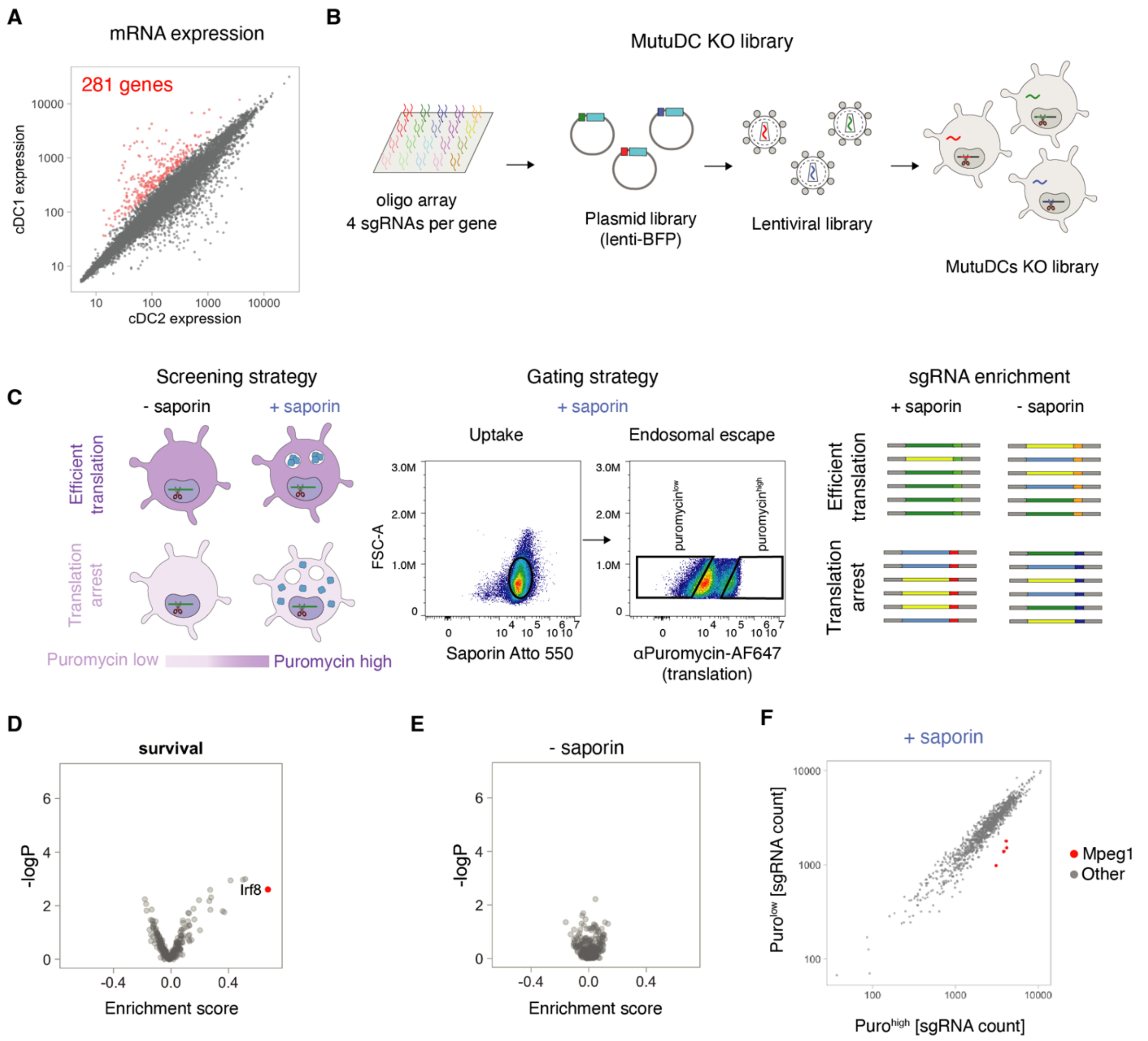

Fig S3. Genetic screen identifies Mpeg1 as a candidate regulator of endocytic escape in DCs.

(A) cDC1 and cDC2 transcript comparison (data from the ImmGen consortium). 281 genes (coloured in red) met the log2(cDC1/cDC2) > 1.3 cut-off and were included in the CRIPSR/Cas9 library (*32*).

(B) The CRISPR/Cas9 library consisted of 4 sgRNAs per gene in a BFP-expressing lentiviral vector. Cas9-expressing MutuDCs were transduced with at an MOI of 0.3. Cells were sorted for BFP expression and expanded.

(C) Schematic representation of the CRISPR/Cas9 screen. For the screen, the saporin-puromycin assay was performed with a 2 h pulse of 0.5 mg/mL saporin (11:1 ratio of unlabelled to Atto550-labelled saporin). Cells were first gated on Atto550 to control for uptake efficiency and split into two bins: puro^{high} (saporin escape) or puro^{low} (saporin retention). Puro^{high} and puro^{low} cells were also collected in the absence of saporin to identify guides that might have a global effect on translation. Genomic DNA was then isolated and prepared for next generation sequencing of the sgRNAs.

(D) Volcano plot showing the sgRNAs enrichment analysis for the MutuDC library relative to the starting plasmid library. Each of the dots represents one targeted gene. Guides against *Irf8* were depleted from the cell MutuDC cell library, in line with the role of Irf8 in survival of terminally differentiated cDC1s (*62*).

(E) Volcano plots showing the sgRNAs enrichment analysis for the control screen in the absence of saporin. Each of the dots represents one targeted gene. Data represent the combined mean enrichment scores and the non-adjusted p values from three independent experiments (Fisher's method).

(F) Relative abundance of sgRNAs in purohigh and purolow populations from the saporin-puromycin-based genetic screen. The screen was performed in three biological repeats. The sgRNA counts from each population in each screen replicate were normalised, and the average of the counts for purohigh and purolow populations is plotted. Each dot corresponds to one sgRNA. The sgRNAs targeting Mpeg1 are highlighted in red.

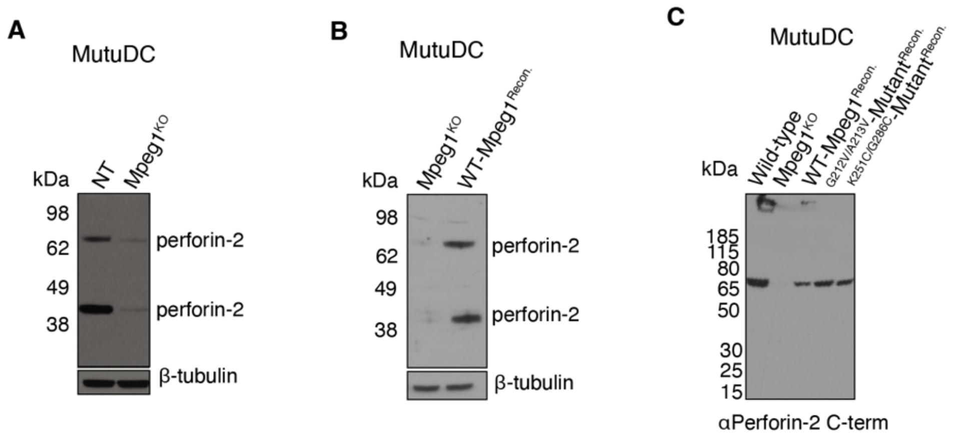

Fig S4. Characterization of the *Mpeg1*^{KO} and rescue MutuDC lines.

(A) Generation of the *Mpeg1*^{KO} MutuDC line. Please note the MutuDCs transduced with sgRNAs were sorted for BFP+ cells, but not clonally cloned. Perforin-2 protein levels in *Mpeg1*^{KO} and NT MutuDCs were assessed by Western blot under reducing conditions. β-tubulin was used as a loading control.

(B) Perforin-2 protein levels in *Mpeg1*^{KO} and *Mpeg1*^{KO} MutuDCs complemented with sgRNA resistant *Mpeg1* were assessed by Western blot under reducing conditions. β-tubulin was used as a loading control.

(C) Protein levels of WT and perforin-2 mutants in reconstituted *Mpeg1*^{KO} MutuDCs were assessed by Western blot under non-reducing conditions.

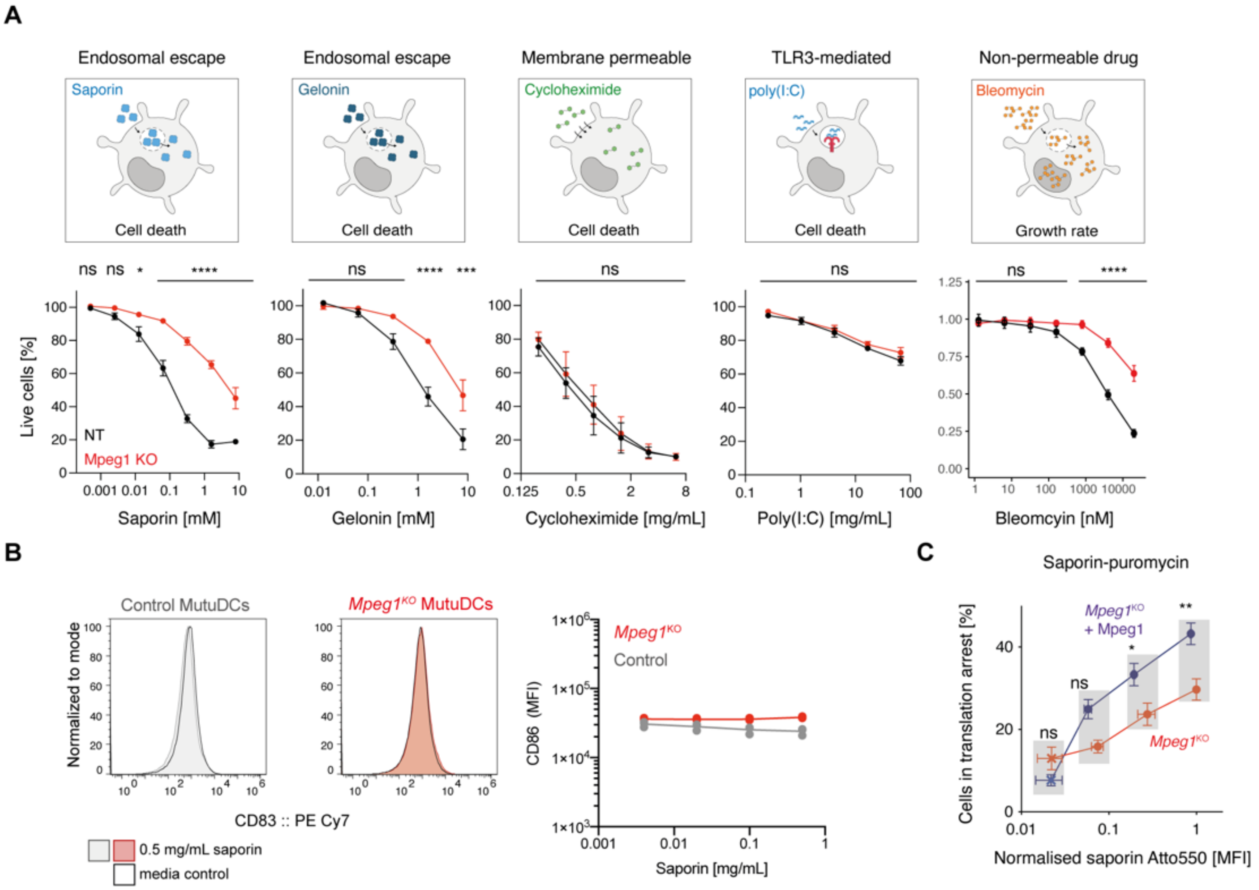

Fig S5. Perforin 2 facilitates endocytic escape of diverse cargo in MutuDCs

(A) *Mpeg1*^{KO} and NT MutuDCs were treated for 24 h with either saporin, gelonin or cycloheximide or for 48 h with HMW-Poly(I:C). Cells were stained with a fixable live/dead stain, and viability was assessed by flow cytometry. For bleomycin, the cells were plated in the presence or absence of bleomycin and cultured in an Incucyte® for 48 h to monitor the growth rate. Data represent mean and SEM of three independent experiments, ns, not significant; **P*<0.5; ***P*<0.01; ****P*<0.001; *****P*<0.0001 using a multiple t-test (Bonferroni-Dunn).

(B) Saporin does not activate MutuDCs suggesting it is devoid of endosomal PAMPs. *Mpeg1*^{KO} and NT MutuDCs were pulsed with saporin for 3 h at 37°C. MutuDC activation was monitored by flow cytometry. Histograms are representative for three independent experiments. Data represents CD86 MFI from three independent experiments.

(C) Quantification of translation arrest in Mpeg1KO and Mpeg1-complemented Mpeg1KO MutuDCs (see also fig. S4B). Cells were pulsed with saporin (11:1, unlabelled:Atto 550-labelled saporin) for 2 h, and translation was monitored by a 30 min puromycin chase. The x-axis represents Atto 550 MFI, normalised to Mpeg1KO MutuDCs Atto 550 MFI at the highest saporin concentration. Data represent mean and SEM of three independent experiments, ns, not significant; *P<0.5; ** P<0.01; *** P<0.001; ****P<0.0001 using a multiple unpaired t-test (two-stage step-up, Benjamini, Krieger and Yekutieli). Significance symbols in the plot refer to the differences in proportion of cells in translation arrest. Differences in saporin-Atto 550 MFI were not significant.

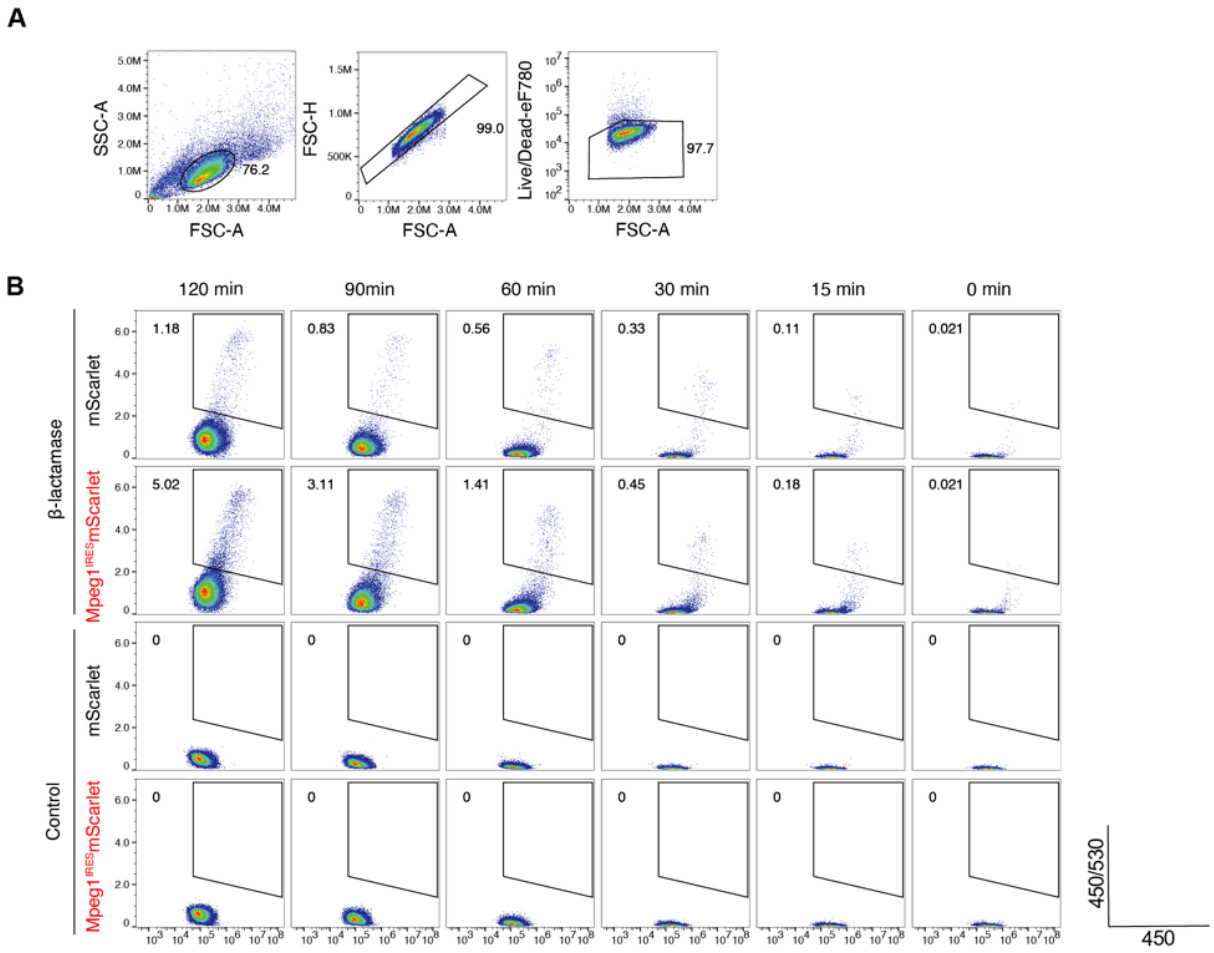

Fig S6. Gating strategy for the CCF4 β-lactamase assay.

(A) Flow cytometry gating strategy for the CCF4 β-lactamase assay in mScarlet and Mpeg1^{IRES}-mScarlet HeLa cells.

(B) Representative gating for CCF4 cleavage for each condition. Gates were defined to exclude a population with spontaneous conversion of CCF4 in the absence of β-lactamase over time.

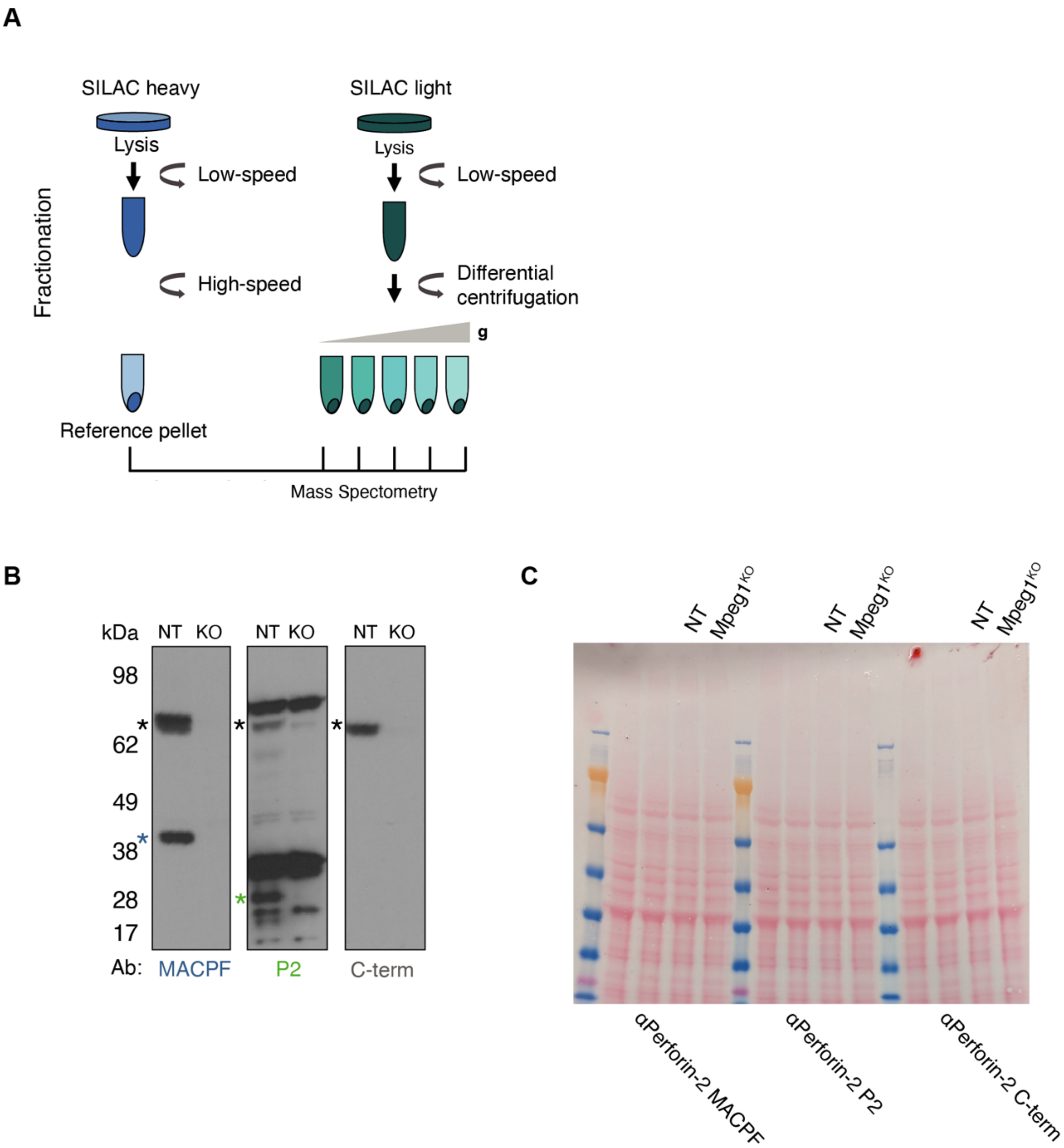

Fig S7. Assessing the proteolytic cleavage of perforin-2.
(A) Schematic representation of fractionation-based mass spectrometry for organellar mapping in MutuDCs. A SILAC heavy reference pellet is generated by spinning cells at a high speed and is then spiked into the light fractions. SILAC light cells are centrifuged at a range of speeds to partially separate organelles. The abundance of proteins across the fractions can then be determined by quantitative mass spectrometry. This figure has been reproduced from (*19*) and is licenced under CC BY 4.0.

(B, C) Analysis of perforin-2 proteolytic maturation. (B) Western blot of NT and *Mpeg1*^{KO} MutuDC lysates under non-reducing conditions using antibodies which recognise different perforin-2 epitopes. (C) Ponceau staining.

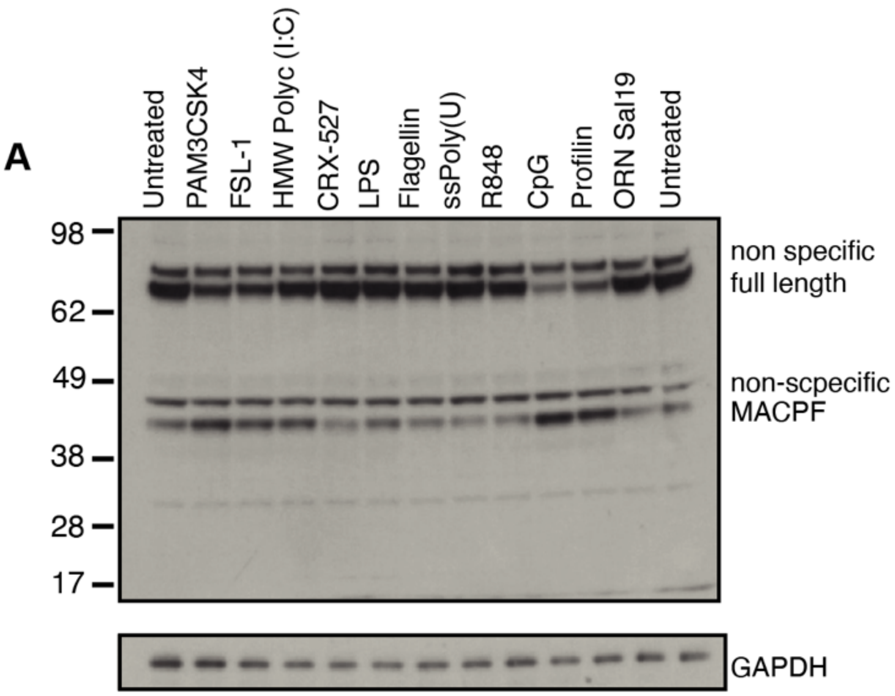

Fig S8. Perforin-2 cleavage is regulated by PAMPs.

(A) MutuDCs were treated with the indicated TLR agonists for 16h. Perforin-2 cleavage was assessed by non-reducing Western blot. GAPDH was used as a loading control. Western blot representative from two independent experiments.

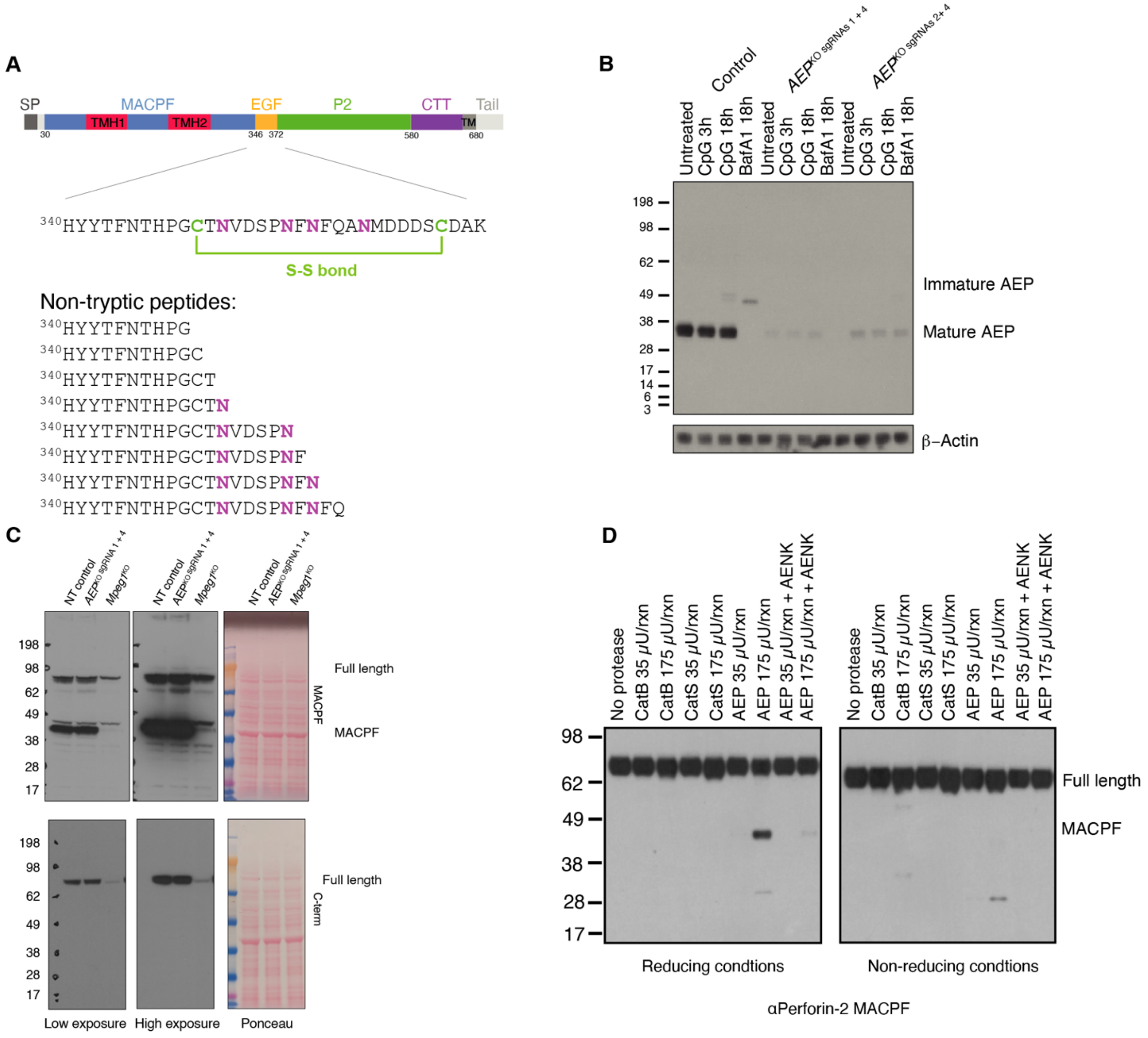

Fig S9. Regulation of perforin-2 cleavage in the EGF domain by AEP.

(A) Schematic representation of perforin-2 highlighting the amino acid sequence within the EGF domain. Sequences of non-tryptic peptides detected in at least two out of three replicates of control cells are listed.

(B) *AEP*^{KO} cells were generated by using sgRNAs listed in Table S4. BFP+ cells were sorted but not clonally selected. Knock out efficiency was assessed by reducing Western blot in untreated and CpG- and BafA1-treated cells. β-Actin was used as a loading control.

(C) Perforin-2 cleavage in control, *Mpeg1*^{KO} and *AEP*^{KO} MutuDCs was analysed by reducing Western blot.

(D) Perforin-2 cleavage by AEP was assessed by reducing and non-reducing Western blot. AEP was preactivated prior to the cleavage reaction by incubation at 37°C for 4 h. Activated AEP was

incubated with purified perforin-2 for 2 h at 37°C in the presence or absence of AEP inhibitor peptide. Following in vitro cleavage, P2 and MACPF domains are linked by the S-S bond (see (A)).

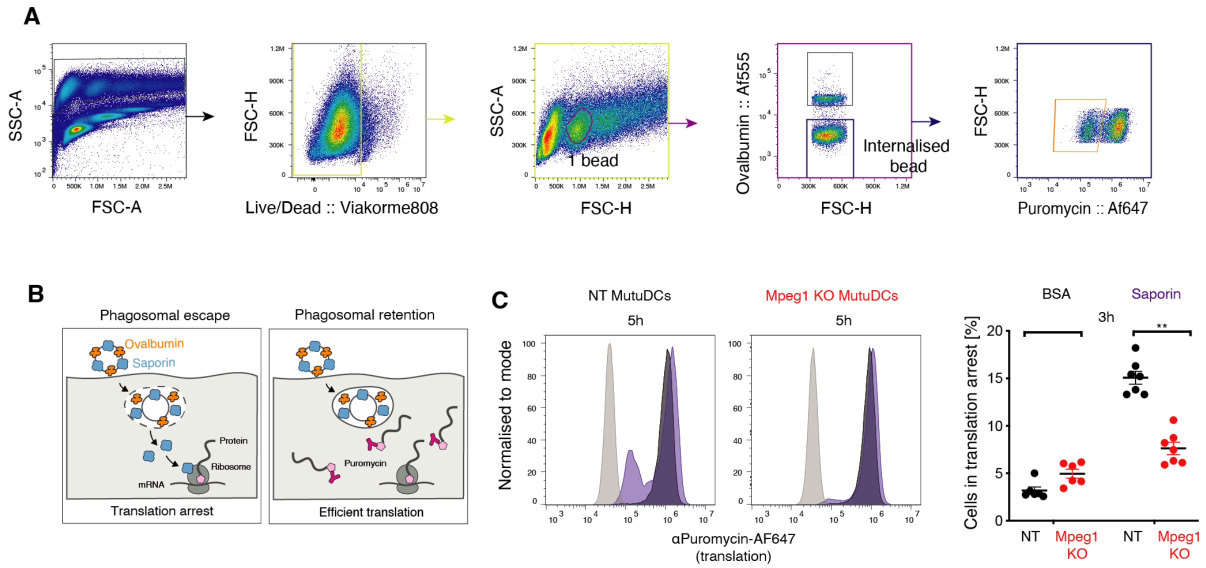

Fig S10. Perforin-2 mediates endocytic escape from antigen-containing phagosomes.

(A) Flow cytometry gating strategy to monitor translation inhibition in MutuDCs containing a single internalised bead.

(B) Schematic representation of the saporin-bead puromycin assay. Cells are pulsed with saporin-conjugated Ova-beads and allowed to internalise them for the indicated time. Translation is then monitored with a 30 min puromycin chase. Incorporated puromycin can then be detected with an αPuromycin antibody and flow cytometry.

(C) *Mpeg1*^{KO} and NT MutuDCs were pulsed for 3 or 5 h with saporin-conjugated Ova-beads and translation was monitored by a 30 min puromycin chase. Histograms are representative of three independent experiments each with two technical replicates. The quantification of translation inhibition represents three independent experiments each with at least two technical replicates, ns, not significant; *P<0.5; **P<0.01; ***P<0.001; ****P<0.0001 using an unpaired t test.

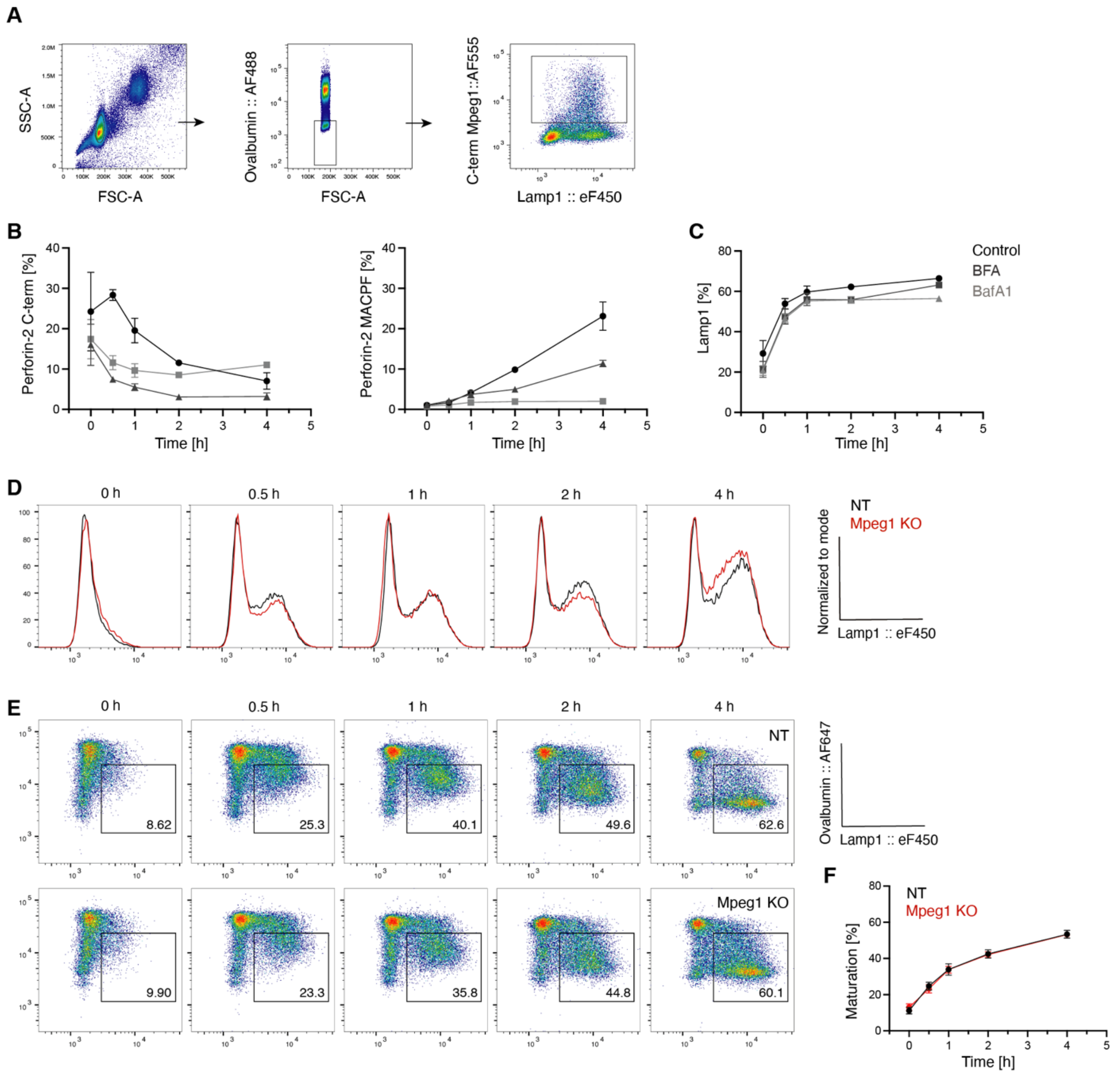

Fig S11. Perforin-2 does not play a role in antigen degradation or phagosome maturation. (A) Flow cytometry gating strategy to identify phagosomes.

(B, C) *Mpeg1*^{KO} and NT MutuDCs were pulsed with Ova-beads and chased for the indicated times in the presence of either BFA or BafA1. Isolated phagosomes were stained with antibodies against (B) perforin-2 with the indicated antibodies and (C) Lamp-1. Data represent mean and SEM of three independent experiments.(D, E, F) *Mpeg1*^{KO} and NT MutuDCs were pulsed with Ova-beads and chased for the indicated times. Isolated phagosomes were stained for Lamp1 and ovalbumin. (D) Lamp1 acquisition. Histograms representative of three independent experiments. (E) Gating to assess phagosome maturation, which is defined as acquisition of Lamp1 and loss (degradation) of ovalbumin. (F) Quantification of phagosome maturation. Mean and SEM of three independent experiments.

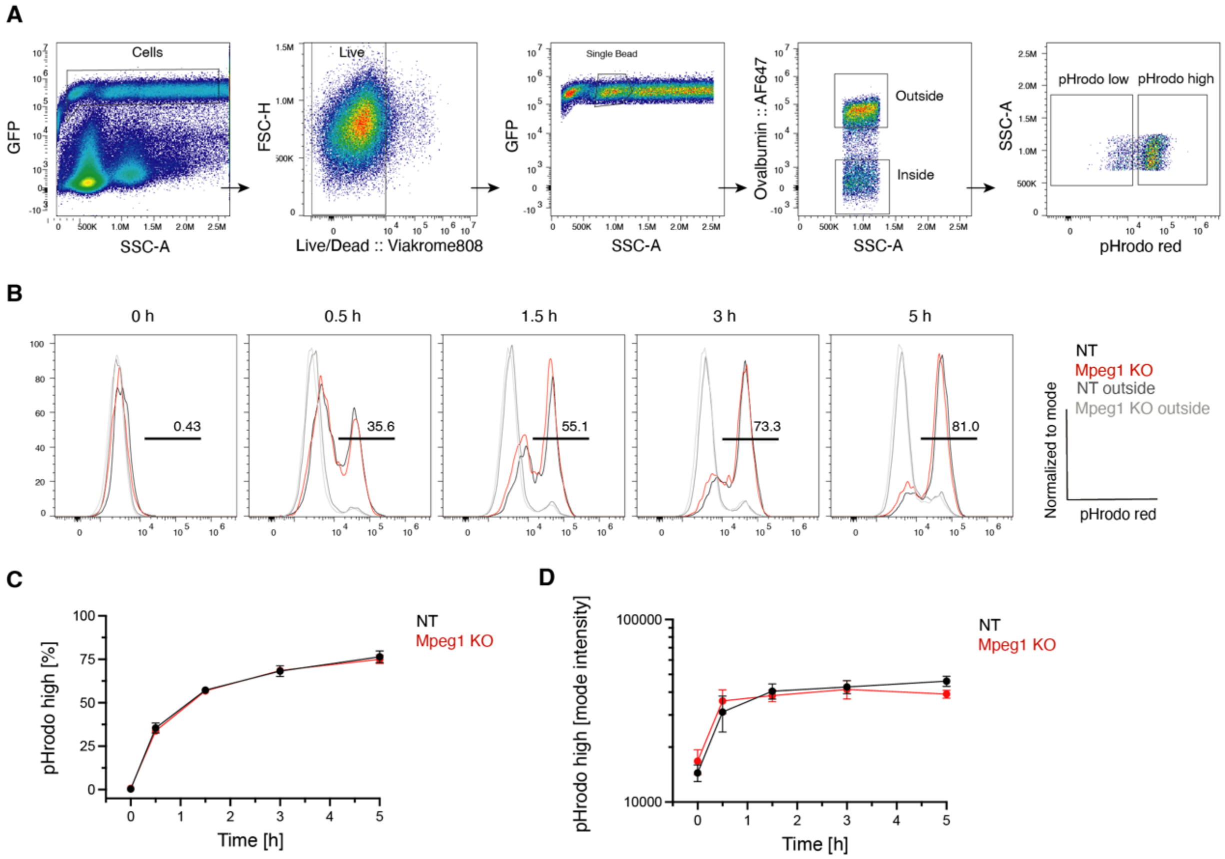

Fig S12. Perforin-2 activity does not alter phagosomal pH.

(A) Flow cytometry gating strategy to identify MutuDCs with a single internalised pHrodo red conjugated Ova-bead.

(B-D) *Mpeg1*^{KO} and NT MutuDCs were pulsed with pHrodo red conjugated Ova-beads and chased for indicated times. Changes in pHrodo red fluorescence were monitored by flow cytometry. (B) Histograms are representative of four independent experiments. (C) Quantification of pHrodo-high MutuDCs and (D) quantification of the mode intensity of the pHrodo-high population as per the gating in (A) representing mean and SEM of four independent experiments.

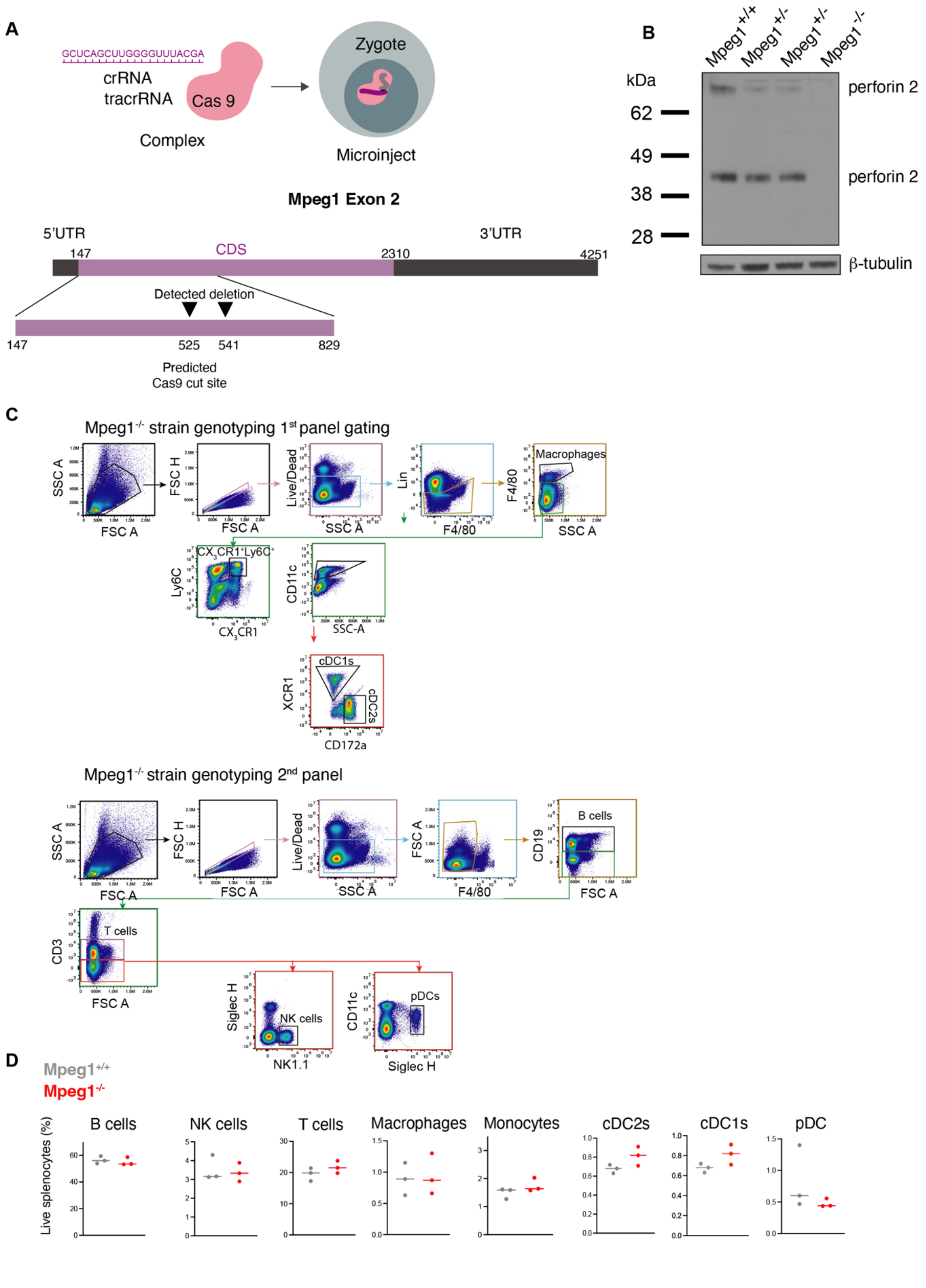

Fig S13. Generation and characterization of Mpeg1-/- mice.
(A) CRISPR/Cas9 strategy for the generation of *Mpeg1* knock-out mice.

(B) Perforin-2 levels in *Mpeg1*$^{+/+}$, *Mpeg1*$^{+/-}$ and *Mpeg1*$^{-/-}$ splenocytes were assessed by Western blot under reducing conditions. β-tubulin was used as a loading control.

(C) Flow cytometry gating strategy for identification of splenic immune cells in *Mpeg1*$^{-/-}$ and *Mpeg1*$^{+/+}$ mice.

(D) Characterization of *Mpeg1*$^{-/-}$ mice. Frequency of B cells, NK cells, T cells, resident and migratory cDC1s and cDC2s, macrophages, monocytes and pDCs. Data represent a single experiment using three mice per genotype.

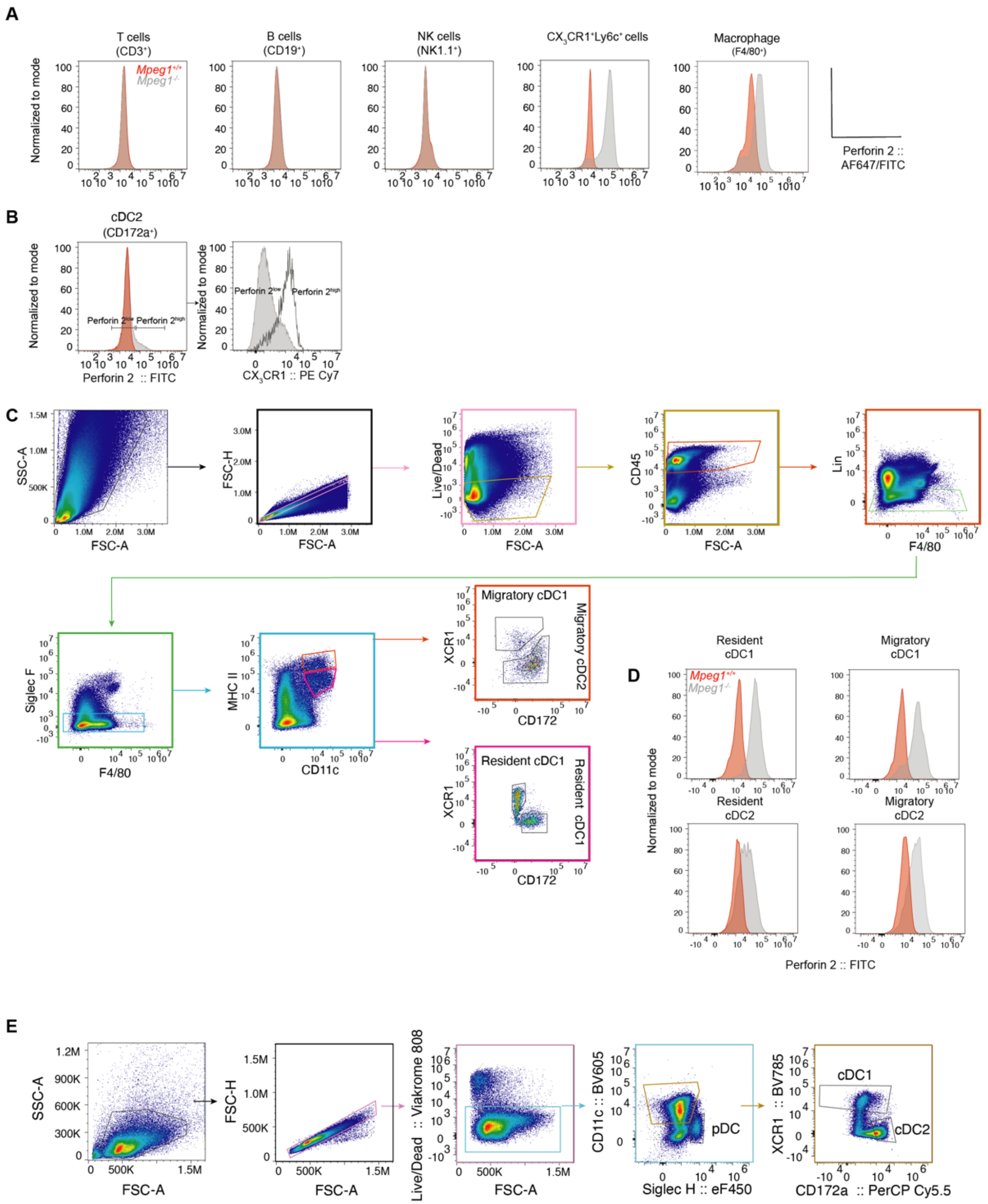

Fig S14. Perforin 2 expression is cell type specific.

(A) Perforin-2 levels in *Mpeg1*$^{+/+}$ and *Mpeg1*$^{-/-}$ in T cells (F4/80$^-$, CD19$^-$, CD3$^-$), B cells (F4/80$^-$, CD19$^+$), NK cells (F4/80$^-$, CD19$^-$, CD3$^-$, NK1.1$^+$), macrophages (Lineage (CD3, CD19, NK1.1)$^-$, F4/80$^+$), $CX_3CR1^+Ly6C^+$ cells (Lineage (CD3, CD19, NK1.1)$^-$, F4/80$^-$, $CX_3CR1^+Ly6C^+$). Histograms are representative for a single experiment using three mice per genotype.

(B) Perforin-2 levels in *Mpeg1*$^{+/+}$ and *Mpeg1*$^{-/-}$ in cDC2s (Lineage (CD3, CD19, NK1.1$^-$, F4/80$^-$, CD11c$^+$, CD172a$^+$) and CX_3CR1 levels in Perforin-2$^+$ and Perforin-2$^-$ cDC2s. Histograms are representative for a single experiment using three mice per genotype.

(C) Flow cytometry gating strategy for identification of lung resident and migratory cDCs.

(D) Perforin-2 levels in *Mpeg1*$^{+/+}$ and *Mpeg1*$^{-/-}$ in resident cDC1s (Lineage (CD3, CD19, NK1.1)$^-$, Siglec F$^-$, F4/80$^-$, CD11c$^+$MHCII$^-$, XCR1$^+$), resident cDC2s (Lineage (CD3, CD19, NK1.1)$^-$, Siglec F$^-$, F4/80$^-$, CD11c$^+$MHCII$^-$, CD172a$^+$), migratory cDC1s (Lineage (CD3, CD19, NK1.1)$^-$, Siglec F$^-$, F4/80$^-$, CD11c$^+$MHCII$^+$, XCR1$^+$), and migratory cDC2s (Lineage (CD3, CD19, NK1.1)$^-$, Siglec F$^-$, F4/80$^-$, CD11c$^+$MHCII$^+$, CD172a$^+$). Histograms are representative for three independent experiments.

(E) Flow cytometry gating strategy for the saporin-puromycin assay with splenic DCs.

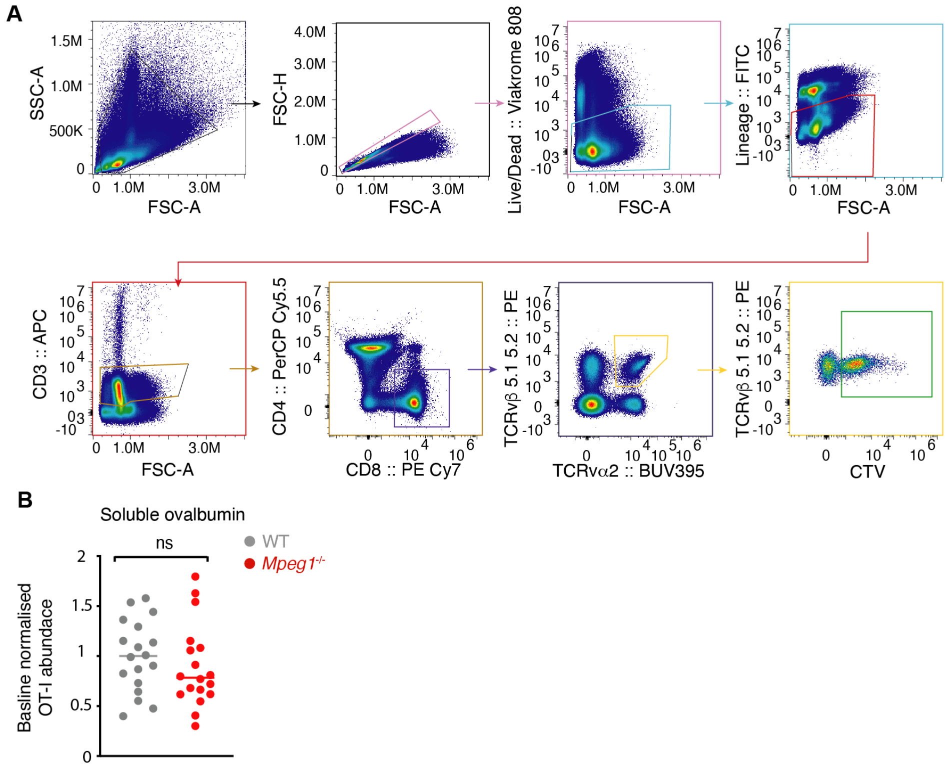

Fig S15. *Mpeg1*$^{-/-}$ mice show no defect in the cross-presentation of soluble antigens.
(A) Flow cytometry gating strategy for identification of OT-I cells.

(B) *Mpeg1*$^{+/+}$ and *Mpeg1*$^{-/-}$ mice were i.v. injected with 0.5x10^{6} CTV-labelled magnetically purified OT-I cells. One day later mice were i.v. injected with 100 µg ovalbumin and 50 µg Poly(I:C). Three days later, OT-I proliferation was assessed by flow cytometry. Data are plotted as individual values normalised to the average wild-type OT-I counts and represent five independent experiments, ns, not significant using an unpaired t-test.

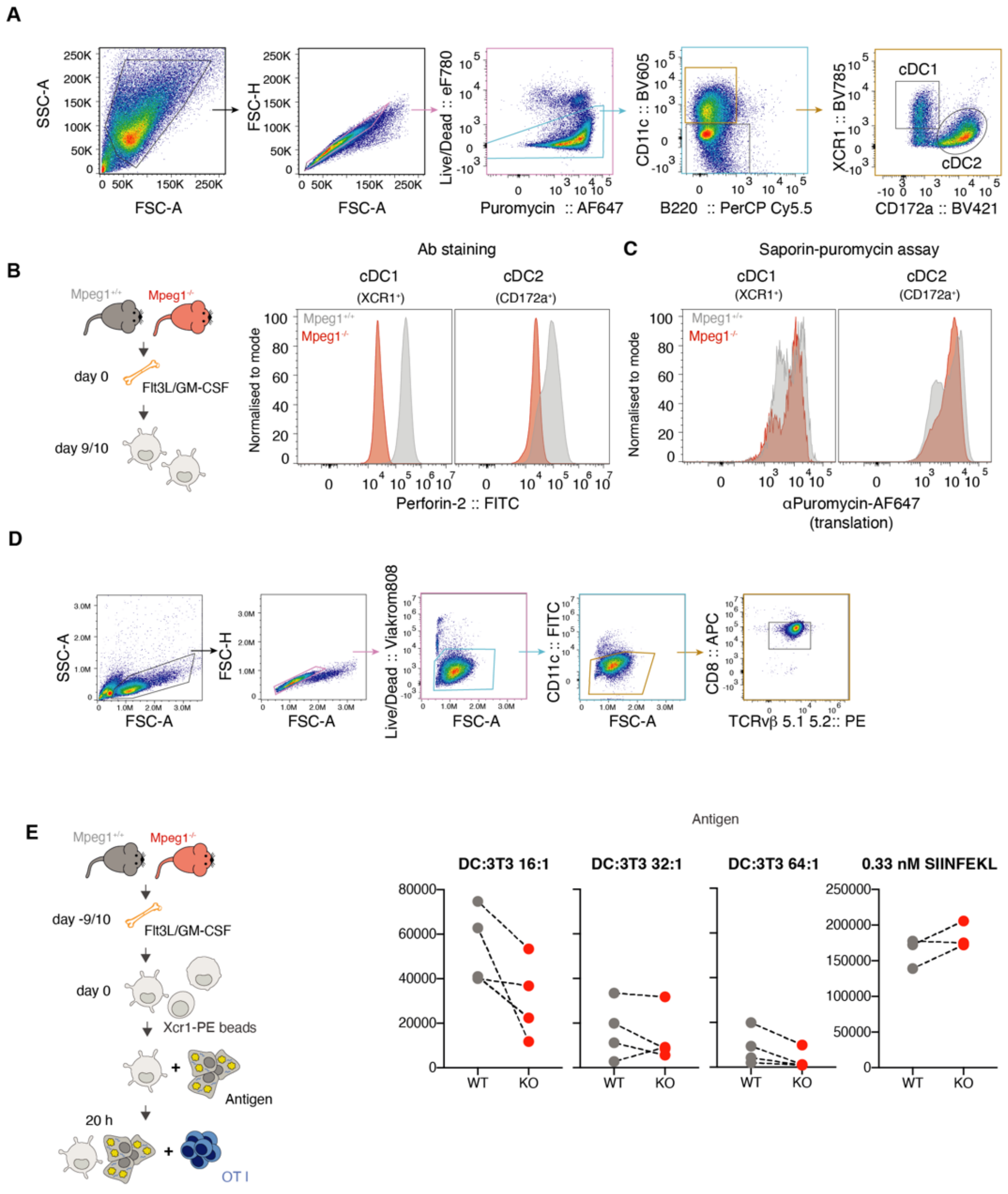

Fig S16. Loss of perforin-2 results in a defect in endocytic escape and cross-presentation of cell associated antigens by bone marrow-derived cDC1s.

(A) Flow cytometry gating strategy for Flt3L/GM-CSF cDC cultures.

(B) Perforin-2 expression was assessed by intracellular staining with an αPerforin-2 antibody and flow cytometry in *Mpeg1*$^{+/+}$ and *Mpeg1*$^{-/-}$ Flt3-L/GM-CSF bone marrow cultures. cDC1s are defined as F4/80^{-}, CD11c^{+}XCR1^{+} and cDC2s as F4/80^{-}, CD11c^{+}CD172a^{+}.

(C) Flt3-L/GM-CSF bone marrow cultures from wild-type and *Mpeg1*$^{-/-}$ mice were pulsed with saporin for 2 h, and translation was monitored by a 30 min puromycin chase. Flt3-L/GM-CSF cDC1s and cDC2s are defined as in (B). Histograms are representative for three independent experiments.

(D) Flow cytometry gating strategy for identification of OT-I cells in the *in vitro* cross-priming assays.

(E) $XCR1^+$ cells from Flt3-L/GM-CSF bone marrow cultures were first pulsed with either UVC irradiated 3T3s coated with ovalbumin and HMW P(I:C) for 20 h or SIINFEKL for 5 h, and then incubated with CTV-labelled purified OT-I cells. Three days later OT-I proliferation was assessed by flow cytometry. Each matching data set corresponds to an independent experiment.

List of Supplementary Tables

Table S1. Genetic screen in MutuDCs.
Data pertaining to the genetic screen in Fig. 2 including immgen gene expression data (cDC1s and cDC2s), a list of the sgRNAs in the custom-made library, sgRNA raw counts, CRISPR/Cas9 screen sample ID and CRISPR/Cas9 screen results.

Table S2. Proteomics data.

(A) Peptide data used for Fig 3A.
Normalised, averaged peptide profiles, and results of the PCA analysis.

(B) Mass spectrometry of CpG- and BafA1-treated MutuDCs and of AEP^{KO} MutuDCs
Proteomics data including protein name, normalised LFQ intensities, p-values (Student's t-test) as well as N-term and C-term cleavage windows.

Table S3. Table of antibodies used in this study.
Information about the antibodies used in this study including clone, fluorochrome and dilutions. See below

Table S4. Table of DNA sequences used in this study.
Details of the plasmids, primers and gene fragments used in this study.

Table S5. Table of reagents used in this study.
See below

Table S3. Table of antibodies used in this study.

Laser	Antibody	Clone	Fluorochrome	Supplier	Catalogue n°	Working concentration
n.a	CD-16/CD32	93	n.a	Thermo Fisher	14-0161-82	1:300
	Lamp1	1D4B	Biotin	Thermo Fisher	13-1071-82	1:200
	C-terminal Mpeg1	Polyclonal	n.a	Cambridge Reasearch Biochemicals	Custom-made	1:500
	MACPF Mpeg1	Polyclonal	n.a	Cloud-Clone	PAB352Mu01	1:200
	Goat Chicken egg albumin	Polyclonal	n.a	OriGene Technologies	AP33373SU-N	1:100
	Mouse chicken egg albumin	TOSGAA1	n.a	BioLegend	520402	1:100
	Rabbit chicken egg albumin	Polyclonal	n.a.	Sigma-Aldrich	C6534	1:500
	Galectin 3	194804	n.a	R&D Systems	mab1154	1:100
	Vps35	A21109	n.a	Santa Cruz	Sc-374372	1:1000
	AEP	Polycloncal	n.a.	R&D Systems	AF2199	1:200
UV	TCRαv2	B20.1	BUV395	BD	743834	1:300
	F4/80	T45-2342	BUV395	BD	565614	1:300
Violet	CD19	1D3	eF450	Invitrogen	48-0193-82	1:300
	F4/80	BM8	BUV395	BioLegend	123131	1:200/1:300
	XCR1	ZET	BV421	BioLegend	148216	1:200/1:300
	CD172a	P84	BV421	BD	740071	1:200/1:300
	VCAM1	429	eF450	Thermo Fisher	48-1061-82	1:200/1:300
	CD11b	M1/70	eF450	Invitrogen	48-0112-82	1:300
	SiglecH	551	PB	BioLegend	129609	1:300
	CD11c	HL3	BV605	BioLegend	117333	1:300
	NK1.1	PK136	Bv650	BioLegend	108735	1:300

	Ly6C	HK1.4	BV785	BioLegend	128041	1:300
	XCR1	ZET	BV785	BioLegend	148225	1:300
	CD11c	HL3	BV786	BD Biosciences	56373	1:300
	Streptavidin	n.a	eF450	Thermo Fisher	48-4317-82	1:1000
Blue	F4/80	BM8	FITC	Thermo Fisher	11-4801-82	1:200/1:300
	NK1.1	PK136	FITC	eBioscience	A14738	1:300
	CD19	1D3	FITC	eBioscience	11-0193-82	1:300
	CD4	RM4-5	PerCP-Cy5.5	eBioscience	45-0042-82	1:300
	XCR1	ZET	PerCP-Cy5.5	BioLegend	148208	1:200/1:300
	CD172a	P84	PerCP-Cy5.5	BioLegend	144009	1:200/1:300
	CD11c	N418	PerCP-Cy5.5	BioLegend	45-0114-82	1:300
	Donkey anti-goat	Polyclonal	Af488	Thermo Fisher	A11055	1:1000
YG	TCRvβ5	MR9-4	PE	BD Biosciences	5583190	1:300
	XCR1	ZET	PE	BioLegend	148204	1:300
	Siglec H	551	PE	BioLegend	129605	1:300
	CD86	GL-1	PE	BioLegend	105008	1:300
	CD25	Pc61	PE	BD Biosciences	553866	1:300
	CD64	X54-5/71	PE Cy7	BioLegend	139313	1:200/1:300
	CX3CR1	SA011F11	PE Cy7	BioLegend	149015	1:300
	CD11c	HL3	PE Cy7	BD Biosciences	558079	1:200/1:300
	CD83	Michel-17	PE Cy7	BD Biosciences	121518	1:300
	CD8α	53-6.7	PE Cy7	BioLegend	100721	1:300
	Donkey anti-rabbit	Polyclonal	Af555	Abcam	ab150062	1:1000
Red	Puromycin	12D10	Af647	Sigma-Aldrich	MABE343-AF647	1:200
	CD3	145-211	APC	BD Biosciences	553066	1:300
	CD4	RM4-5	APC	eBioscience	17-0042-83	1:300

	CD8α	53-6.7	APC	BD Biosciences	553035	1:300
	CD64	X54-5/71	APC	BioLegend	139305	1:200
	CD19	6D5	APC	BioLegend	115511	1:300
	CD3	17A2	APC-Cy7	BioLegend	100221	1:300
	CD19	6D5	APC-Cy7	BioLegend	115529	1:300
	NK1.1	PK136	APC-Cy7	BioLegend	108723	1:300
	Fixable Viability Dye	n.a	eF780	Invitrogen	65-0865-14	1:2500
	Donkey anti-mouse	Polyclonal	Af647	Thermo Fisher	A31571	1:500/1:1000
	Donkey anti-rabbit	Polycloncal	Af647	Abcam	ab150075	1:500
Infrared	Fixable Viability Dye	n.a	Viakrome 808	Beckman Coulter	C36628	1:750

Table S5. Table of reagents used in this study.

Item	Cat number	Supplier
Saporin	S9896	Sigma-Aldrich / Merck
Puromycin	A1113803	Thermo Fisher
Gelonin	ALX350-150-M001	Enzo Life Sciences
PBS	14190144	Thermo Fisher
Pierce 20X TBS buffer	28358	Thermo Fisher
IMDM	31980030	Gibco™
Glutamax	12440053	Gibco™
FCS	11550356	Gibco™
HEPES	15630106	Gibco™
β-mercaptoethanol	31350010	Gibco™
Penicillin/streptomycin	11548876	Gibco™
DMEM	31966021	Gibco™
RPMI-1640	6180036	Gibco™
Sodium pyruvate	11360070	Gibco™
Non-essential amino acids	11140050	Gibco™
Red blood cell lysis buffer Hybrid-max	R7757-100ML	Sigma-Aldrich
Valporic acid	P4543-10G	Sigma-Aldrich
Imidazole	56748	Sigma-Aldrich
NaCl	S/3120/63	Fisher Scientific
NaOAc	S/2080/53	Fisher Scientific
Sucrose	1046150	Fisher Scientific
UltraPure 0.5M EDTA pH 8.0	15575020	Thermo Fisher
BSA	A7979	Sigma-Aldrich
Glutaraldehyde	G5882	Sigma-Aldrich
Glycine	G5417	Sigma-Aldrich
CO_2-indepdent medium	18045088	Thermo Fisher
Bafilomycin A1	11038	Cayman Chemical
Brefeldin A	sc-200861C	Santa Cruz Biotechnology
Phenylmethanesulfonyl fluoride (PMSF)	P-7626	Sigma Aldrich
EDTA-free protease inhibitor cocktail	11873580001	Roche
Dithiothreitol (DTT)	D9779	Sigma Aldrich
GM-CSF	130-095-746	Miltenyi
Flat3 Ligand	130-097-372	Miltenyi
Liberase-TL	5401020001	Merck
DNAse I	11284932001	Merck
Collagenase type I	17100-017	Gibco™
Mouse pan-dendritic cell isolation isolation kit	130-1000-875	Miltenyi Biotec

EasySep release mouse PE positive selection kit	17656	Stemcell
EasySep mouse naïve CD8+ T cell isolation kit	19858	Stemcell
Naïve CD8a$^+$ T cell isolation kit	130-096-543	Miltenyi Biotec
Gel extraction kit	28706X4	Qiagen
Gibson Assembly cloning kit	E5510S	NEB
Endura^{TM} competent cells	60242-1	Lucigen
One Shot^{TM} Top10 chemically competent *E.coli*	C404003	Thermo Fisher
Lucigen recovery media	F98226-1	Lucigen
NuncTM square BioAssay dish	166508	Thermo Scientific
Plasmid maxi kit	121162	Qiagen
TransIT-LT1	MIR2304	Mirus
Amicon Ultra-15 centrifugal filters	UFC9003	Amicon
DNeasy blood and tissue kit	69504	Qiagen
Herculase II fusion DNA polymerase	600677	Agilent
Herculase reaction buffer	600677	Agilent
KAPA library quantification kit	KK4828	KAPA Biosystems
Charge switch PCR Clean-up kit	CD12000	Invitrogen
KOD Xtreme hot start DNA	71975	Merck
MluI-HF	R0198S	NEB
NotI-HF	R0189S	NEB
BsmBI	R0739	NEB
CspCI	R0645S	NEB
PspXI	R0656S	NEB
SbfI-HF	R3642-L	NEB
SnaBI	R0130S	NEB
BstXI	R0113L	NEB
BlpI	R0585S	NEB
T4 Polynucleotide Kinase	M0201L	NEB
Alkaline phosphatse, CIP	M0290	NEB
T4 DNA Ligase	M0202	NEB
QIAquick PCR Purification Kit	28104	Qiagen

NEB5α	C2987I	NEB
LDS NuPage	NP0007	Thermo Fisher
Bolt™ reducing agent	NP0009	Thermo Fisher
RIPA	R0278	Thermo Fisher
Tween-20	786-517	G-Bioscience
Triton-X100	BP151-500	Fisher Bioreagents
ECL prime western blotting detection reagent	12316992	Thermo Fisher
μ-slide	80826	Ibidi
GPN	Sc-252858	Santa Cruz Biotechnology
LysoTracker Red DND-99	L7528	Thermo Fisher
CellTrace violet proliferation kit	C34557	Thermo Fisher
Ovalbumin	LS0030540	Worthington
Vaccination grade ovalbumin	Vac-pova	InvivoGen
HMW Poly(I:C)	Tlrl-pic	InvivoGen
PAM3CSK4	Tlrl-pm2s-1	InvivoGen
FSL-1	Tlrl-fsl	InvivoGen
CRX-527	Tlrl-crx527	InvivoGen
LPS	Vac-3pelps	InvivoGen
Flagellin	Tlrl-bsfla	InvivoGen
ssPoly(U)	Tlrl-sspu	InvivoGen
R848	Tlrl-r848	InvivoGen
CpG ODN2395	Tlrl-2395	InvivoGen
Profilin	AG-40B-0121-C010	AdipoGen Life Sciences
ORN Sa19	Tlrl-orn19	
Pierce BCA protein assay kit	23227	Thermo Scientific
LysC	12--02541	Wako Chemicals
Trypsin	T6567	Sigma-Aldrich
SDB-RPS	66886-U	Sigma-Aldrich
Polyethyleneimine Max	49553-93-7	Polysciences
Asparagine Endopeptidase	2058-CY-010	R&D Systems
AEP fluorgenic substrate	I-1865	Bachem
AEP inhibitor peptide	53379	Sigma-Aldrich
Nano-Glo® live cell assay system	N2011	Promega
PrestoBlue viability reagent	A13261	Invitrogen
Polybead® Amino Microspheres 3.00μm	17145-5	Polysciences

Atto 550 protein labelling Kit	51146-1KT	Thermo Fisher
Zeba Spin 7K MWCO Desalting Columns	10415545	Thermo Fisher
SPDP (succinimidyl 3-(2-pyridyldithio)propionate)	21857	Thermo Fisher
Traut's reagent (2-iminothiolane)	26101	Thermo Fisher
pHrodo iFL Red STP ester, amin reactive dye	P36011	Thermo Fisher
BD Cytofix/Cytoperm™ Kit	554714	BD Biosciences
Cas9 nuclease	CP01	PNA Bio
RNAase free TrisHCl	T2444-100mL	Sigma-Aldrich
RNAase free EDTA	AM9260G	Thermo Fisher
tracrRNA	TRACRRNA05M-5NMOL	Sigma-Aldrich
